# Photocatalytic Dehalogenative Deuteration of Halides over a Robust Metal–Organic Framework

**DOI:** 10.1002/anie.202306267

**Published:** 2023-10-26

**Authors:** Tian Luo, Zi Wang, Yinlin Chen, Hengzhao Li, Mengqi Peng, Floriana Tuna, Eric J. L. McInnes, Sarah J. Day, Jie An, Martin Schröder, Sihai Yang

**Affiliations:** ^1^ Department of Chemistry University of Manchester Manchester M13 9PL UK; ^2^ Department of Nutrition and Health China Agricultural University Beijing 100193 China; ^3^ Photon Science Institute University of Manchester Manchester M13 9PL UK; ^4^ Diamond Light Source Harwell Science Campus Oxfordshire OX11 0DE UK; ^5^ College of Chemistry and Molecular Engineering Beijing National Laboratory for Molecular Sciences Peking University Beijing 100871 China

**Keywords:** Dehalogenative Deuteration, EPR, Metal–Organic Frameworks, Photocatalysis, SXPD

## Abstract

Deuterium labelling of organic compounds is an important process in chemistry. We report the first example of photocatalytic dehalogenative deuteration of both arylhalides and alkylhalides (40 substrates) over a metal–organic framework, MFM‐300(Cr), using CD_3_CN as the deuterium source at room temperature. MFM‐300(Cr) catalyses high deuterium incorporation and shows excellent tolerance to various functional groups. Synchrotron X‐ray powder diffraction reveals the activation of halogenated substrates via confined binding within MFM‐300(Cr). In situ electron paramagnetic resonance spectroscopy confirms the formation of carbon‐based radicals as intermediates and reveals the reaction pathway. This protocol removes the use of precious‐metal catalysts from state‐of‐the‐art processes based upon direct hydrogen isotope exchange and shows high photocatalytic stability, thus enabling multiple catalytic cycles.

## Graphical abstract

The metal–organic framework MFM‐300(Cr) acts as a photocatalyst for the dehalogenative deuteration of both arylhalides and alkylhalides (40 substrates) with high deuterium incorporation. Synchrotron PXRD reveals the activation of halogenated substrates via confined binding within MFM‐300(Cr). In situ EPR spectroscopy confirms the formation of carbon‐based radicals as intermediates and reveals the reaction pathway.

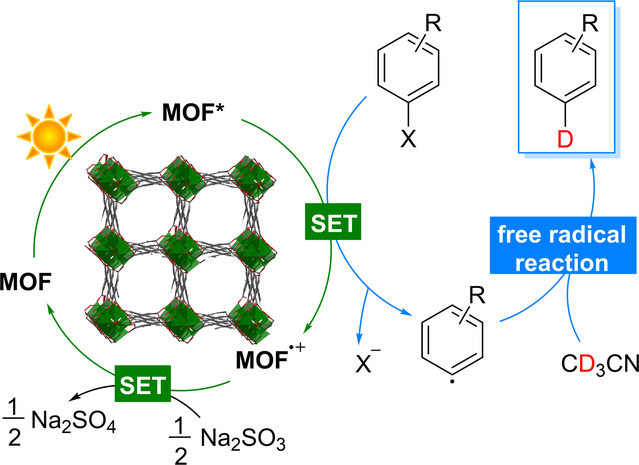

## Introduction

**Figure 1:**
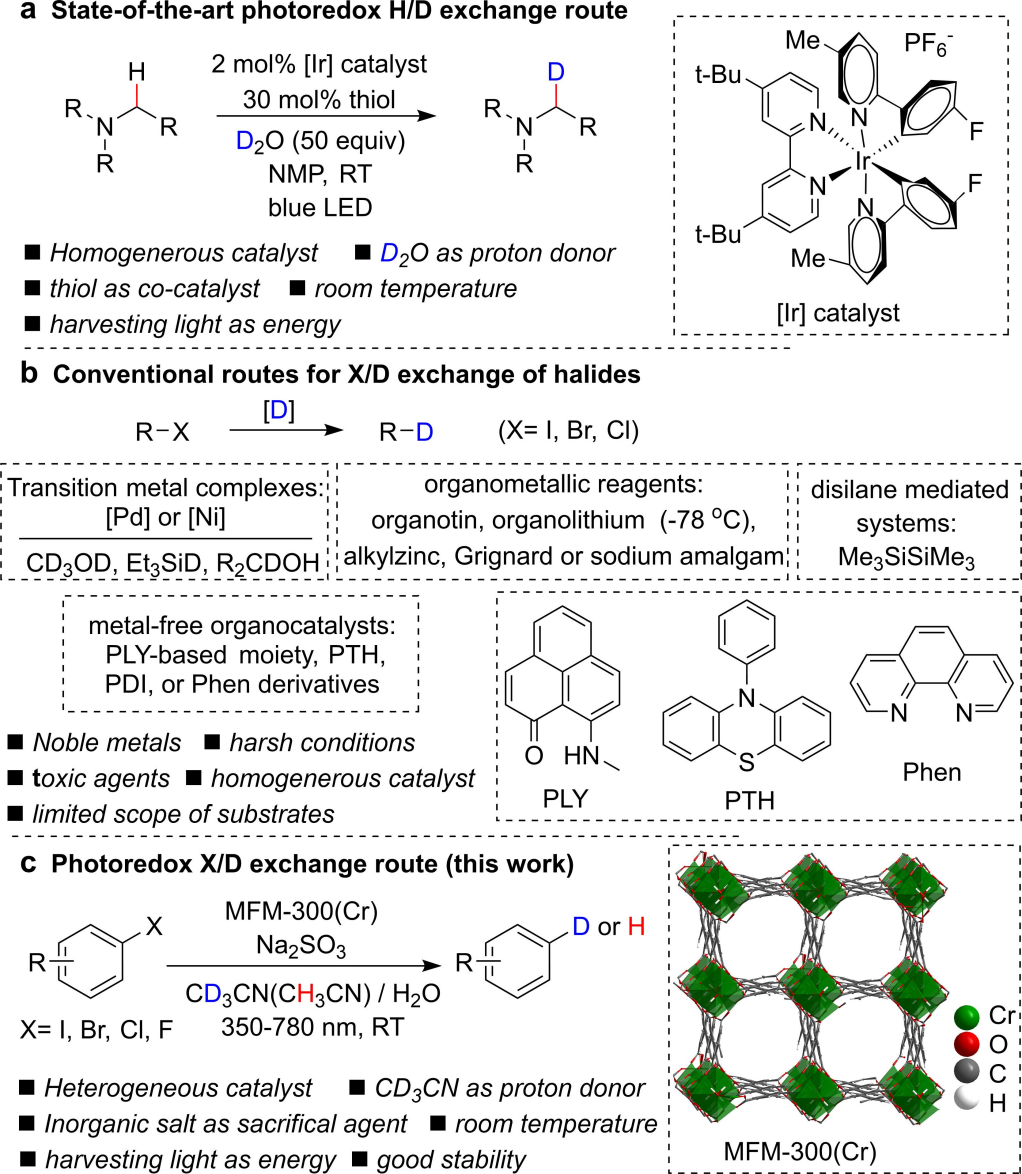
Routes to deuterium labelling of organic compounds. (a) State‐of‐the‐art photo‐redox H/D exchange route catalysed by an [Ir] complex. (b) X/D exchange of halides using transition metal complexes, organometallic regents, disilane mediated systems and metal‐free organocatalysts (PLY=hydrocarbon phenalenyl derivatives, PTH=10‐phenylphenothiazine, PDI=perylenediimide, Phen=1,10‐phenanthroline and phenothiazine derivatives). (c) Photo‐redox route catalysed by MFM‐300(Cr).

Deuterium labelling of organic compounds is employed widely in organic chemistry and the pharmaceutical industry,[^1^, ^2^, ^3^] and the state‐of‐the‐art methoology is based upon direct hydrogen isotope (H/D) exchange. However, this often suffers from low deuterium incorporation and regioselectivity.[^4^, ^5^, ^6^] Moreover, homogeneous catalysts based upon precious metals (primarily Ir‐complexes, Figure 1a) or strong acid/base and high temperatures are often required for the activation of inert C−H bonds. This places restrictions to the tolerance of functional groups and the scope of substrates that can converted.[^7^, ^8^, ^9^] Dehalogenative deuteration of organic halides emerges as a promising alternative method for deuterium labelling with high regioselectiveity, and a wide range of catalysts have been investigated for C−X/C−D exchange, including transition metal complexes (Pd−NHC),[^10^, ^11^] organometallic regents (Bu_3_SnH),[^12^, ^13^, ^14^] metal‐free organocatalysts (Figure 1b),[^15^, ^16^, ^17^, ^18^] semiconductors (CdSe)^[19]^ and disilane (Me_3_SiSiMe_3_) mediated systems.^[20]^ The development of efficient, non‐toxic processes using non‐precious metal catalysts to afford high site‐selectivity, deuterium incoporation and catalytic stability under mild conditions remains a challenge.

Possessing high porosity, structural diversity and semiconductor‐like behaviour, metal–organic framework (MOF) materials have been reported as photocatalysts for CO_2_ reduction,^[21]^ water splitting,^[22]^ and organic redox reactions.^[23]^ MOF‐based photocatalysts operate via a three‐step process^[24]^ involving light absorption and photo‐induced charge‐hole separation; migration of photogenerated electrons and holes to reactive sites through ligand‐to‐metal charge‐transfer (LMCT) or metal‐to‐ligand charge‐transfer (MLCT); half‐reaction of oxidation or reduction with redox equivalents. Thus, the precise mechanism of electron transfer often plays an important role in determining the overall catalytic performance. Furthermore, as reported for supramolecular cages[^25^, ^26^, ^27^] and zeolites,[^28^, ^29^] host–guest interactions between the porous scaffolds and adsorbed substrates have significant impact on the activation of substrates and thus the catalytic activity. We have recently found that the formation of hydrogen bonds between the metal‐hydroxyl groups immobilised in a MOF catalyst and adsorbed ketone/aldehyde compounds can effectively promote the photo‐reduction of carbonyl groups.^[30]^ Moreover, the host–guest interaction can also facilitate the transfer of photo‐induced electrons, thus mitigating electron‐hole recombination and promoting the catalytic efficiency.^[31]^ However, deuterium labelling of organic compounds over MOF‐based photocatalysts has not been reported to date.

Here, we report the efficient photocatalytic dehalogenative deuteration of a broad range of aryl‐ and alkyl‐ halides over the robust material MFM‐300(Cr) under visible light (350–780 nm) irradiation, with CD_3_CN and Na_2_SO_3_ acting as deuterium source and sacrificial agent, respectively. High deuterium incorporation and excellent regioselectivity have been achieved at room temperature for 40 substrates, including 25 iodides and 15 bromides. The confined binding of halogenated substrates via formation of host–guest π⋅⋅⋅π interactions and hydrogen bonding within the MOF pores has been confirmed by synchrotron X‐ray powder diffraction (SXPD) of substrate‐loaded MFM‐300(Cr). In situ electron paramagnetic resonance (EPR) spectroscopy reveals the generation of de‐halogenated carbon radicals as the reaction intermediate to drive the photocatalytic process, and a reaction pathway proposed.

## Results and Discussion

MFM‐300(Cr), [Cr_2_(OH)_2_(L)] (H_4_L=biphenyl‐3,3′,5,5′‐tetracarboxylic acid), comprises chains of corner sharing [Cr(OH)_2_O_4_] octahedra linked by mutually *cis*‐μ_2_‐OH groups, which are further bridged by tetracarboxylate L^4−^ ligands.^[32]^ This arrangement generates channels bounded by {Cr−O(H)−Cr} groups and phenyl rings in a ‘wine rack’ array. Scanning electron microscopy (SEM) images of MFM‐300(Cr) show cuboid‐shaped crystals with an average size of ca. 400 nm (Figure S3a). Desolvated MFM‐300(Cr) exhibits a Brunauer–Emmett–Teller (BET) surface area of 1147 m^2^ g^−1^ (Figure S4). The solid‐state UV/Vis diffuse reflectance spectrum (UV‐DRS) of MFM‐300(Cr) shows a wide window of light absorption up to 800 nm with two absorption bands in the visible light region centred at 417 and 588 nm (Figure S5). The narrowest optical band gap (E_g_) of MFM‐300(Cr) is determined to be 1.77 eV, which is comparable to the leading MOF‐based photocatalysts such as NH_2_−MIL‐125(Ti) (2.60 eV),^[33]^ NH_2_−UiO‐66(Zr) (2.75 eV)^[34]^ and NH_2_−MIL‐101(Fe) (1.77 eV).^[35]^

**Figure 2:**
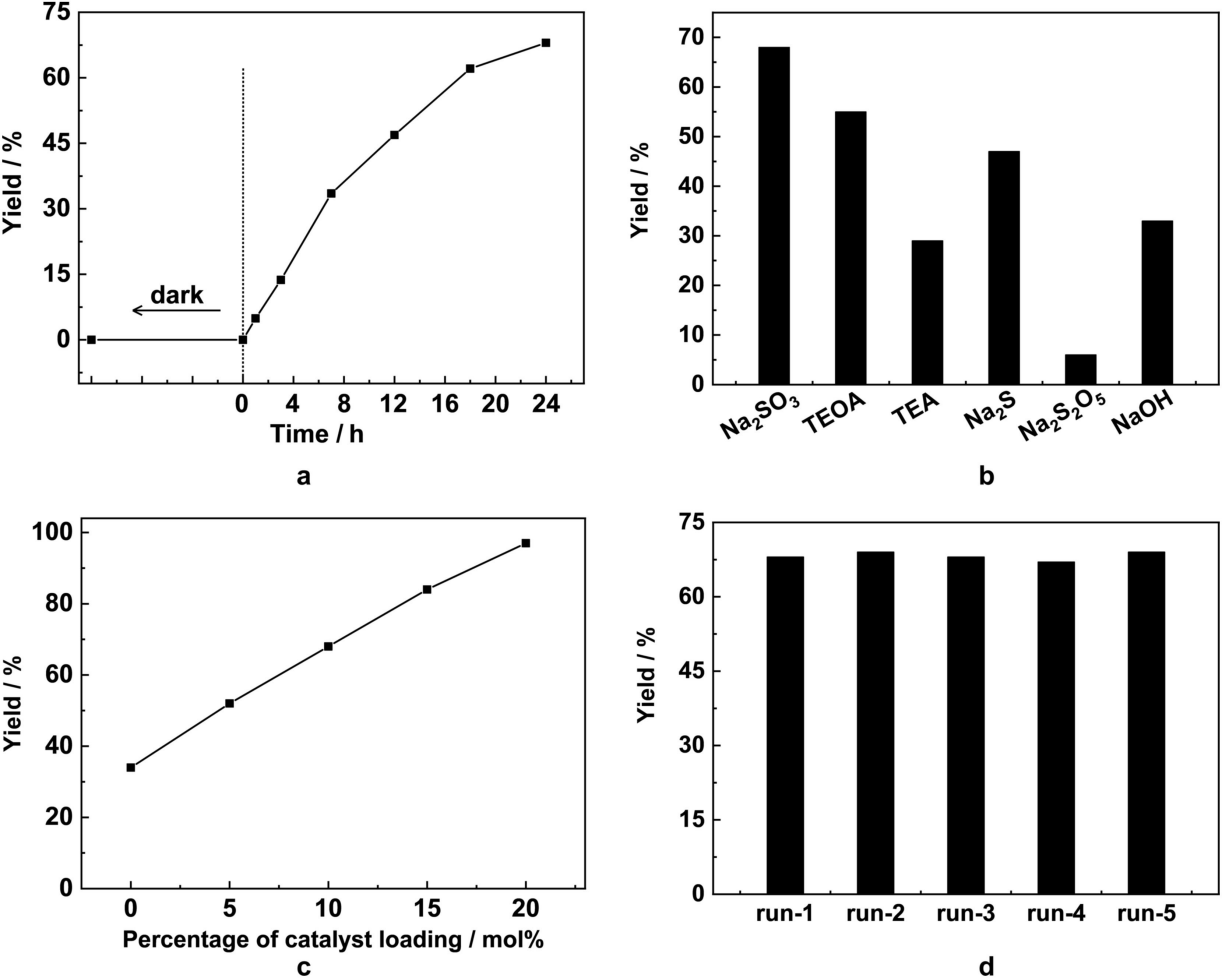
Optimisation of photocatalytic dehalogenation of 4‐iodoanisole over MFM‐300(Cr). The effect of (a) reaction time; (b) reducing agents; (c) catalyst loading amount; (d) recycling test and yield of product on each cycle. Reaction conditions for a typical experiment: substrate (0.50 mmol), MFM‐300(Cr) (10 mol%, 0.05 mmol), CH_3_CN/H_2_O (15 mL/15 mL), Na_2_SO_3_ (0.5 M), 25 °C, 350–780 nm (a 300 W Xe lamp), light irradiation for 24 h. The amounts of salts in (b): TEOA (3.0 mL, 22.6 mmol), TEA (3.0 mL, 21.5 mmol), Na_2_S or Na_2_S_2_O_3_ (15.0 mmol), NaOH (0.075 mmol, pH=11.7).

The hydrodehalogenation of 4‐iodoanisole (C−X/C−H exchange) was chosen as a model reaction to optimise the reaction conditions and to monitor the effects of reaction time, reducing agent and loading of MFM‐300(Cr) catalyst. The yield of anisole increased with increase of reaction time and reached 68 % at 24 h with a 10 mol% catalyst loading (Figure 2a). When the catalyst loading was increased to 20 mol%, a high yield of 97 % was obtained after 24 h (Figure 2c). Triethanolamine (TEOA) and triethylamine (TEA) are employed widely as sacrificial electron donors in photocatalysis,[^36^, ^37^] however, these organic amines exhibit poor stability upon light irradiation. A variety of reductants including TEOA, TEA and inorganic salts (Na_2_SO_3_, Na_2_S, Na_2_S_2_O_5_ and NaOH) were tested with a catalyst loading of 10 mol% for 24 h (Figure 2b). Na_2_SO_3_ gave the best yield of 68 %, exceeding that of TEOA and TEA (55 % and 29 %, respectively). A biphasic system comprising of H_2_O and CH_3_CN was employed to dissolve Na_2_SO_3_ and the organic substrate, respectively, and, powder X‐ray diffraction (PXRD) analysis confirmed that Na_2_SO_3_ was partially oxidised to Na_2_SO_4_ (Figure S6). To gain further insight into this reaction, a set of control experiments were conducted (Table S1). In the absence of MFM‐300(Cr), only a low yield of 34 % for anisole was observed. Replacing MFM‐300(Cr) with an equivalent amount powdered mixture of CrCl_3_ ⋅ H_2_O and H_4_L also resulted in a low yield of 34 %. These results identified the role of MFM‐300(Cr) as the photocatalyst and the importance of its framework structure. In the absence of Na_2_SO_3_, with or without MFM‐300(Cr), less than 7 % yield of anisole was observed. Under dark conditions, no product was formed confirming the photocatalytic nature of this reaction. Moreover, in the absence of H_2_O or reducing the concentration of Na_2_SO_3_ results in reduced yields of 12 % and 48 %, respectively. Thus, the reaction conditions were optimised for 0.5 mmol of substrate at a catalyst loading of 10–20 mol%, 0.5 M of Na_2_SO_3_ in CH_3_CN/H_2_O for 24 h. Importantly, MFM‐300(Cr) could be recycled readily and retained its catalytic activity for at least five cycles with only minor change in the structure (Figure 2d and Figure S2a).

To investigate the source of deuterium, a set of control experiments were conducted in different solvents using 4‐iodoanisole and 4′‐bromoacetophenone as the iodo and bromo substrates, respectively (Table 1). The reaction of 4‐iodoanisole gives a C−I/C−H exchange yield of 68 % in CH_3_CN/H_2_O and a C−I/C−D exchange yield of 66 % with a yield for deuterium exchange of 95 % in CD_3_CN/D_2_O. Interestingly, C−I/C−H exchange was observed in CH_3_CN/D_2_O with no detectable amount of deuterium incorporated, and C−I/C−D exchange in CD_3_CN/H_2_O shows a yield for deuterium exchange of 92 %, indicating that the deuterium source for this reaction is CD_3_CN rather than D_2_O. The same result was observed for 4‐bromoacetophenone, for which C−Br/C−H exchange was observed in CH_2_Cl_2_/H_2_O and in CH_2_Cl_2_/D_2_O, and C−Br/C−D exchange in CD_2_Cl_2_/H_2_O and in CD_2_Cl_2_/D_2_O, confirming CD_2_Cl_2_ as the deuterium source. These control experiments confirm that organic solvents rather than water serve as the deuterium source in this protocol, which is distinct to the examples reported in the literature,[^19^, ^38^] reflecting the strong adsorption of organic solvents by the MOF. For example, when porous inorganic CdSe nanosheets were employed as photocatalyst for deuterodehalogenation of aryl halides in CH_3_CN/D_2_O, D_2_O splitting occurs to supply deuterium free radicals to drive the reaction.^[19]^

**Table 1:**
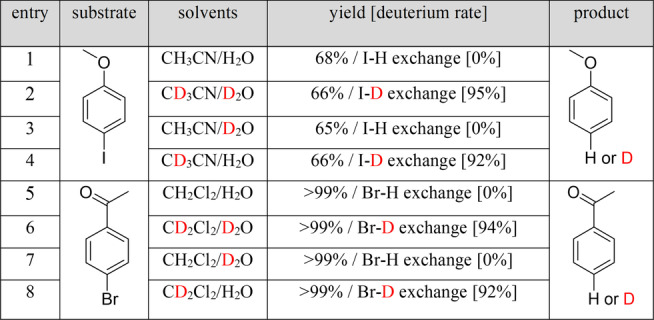
Control experiments for mechanistic studies on the deuterium source.

Reaction conditions: Substrate (0.50 mmol), MFM‐300(Cr) (10 mol%, 0.05 mmol), organic solvent/H_2_O (15 mL/15 mL), Na_2_SO_3_ (0.5 M), 25 °C, 350–780 nm, 24 h.

**Figure 3:**
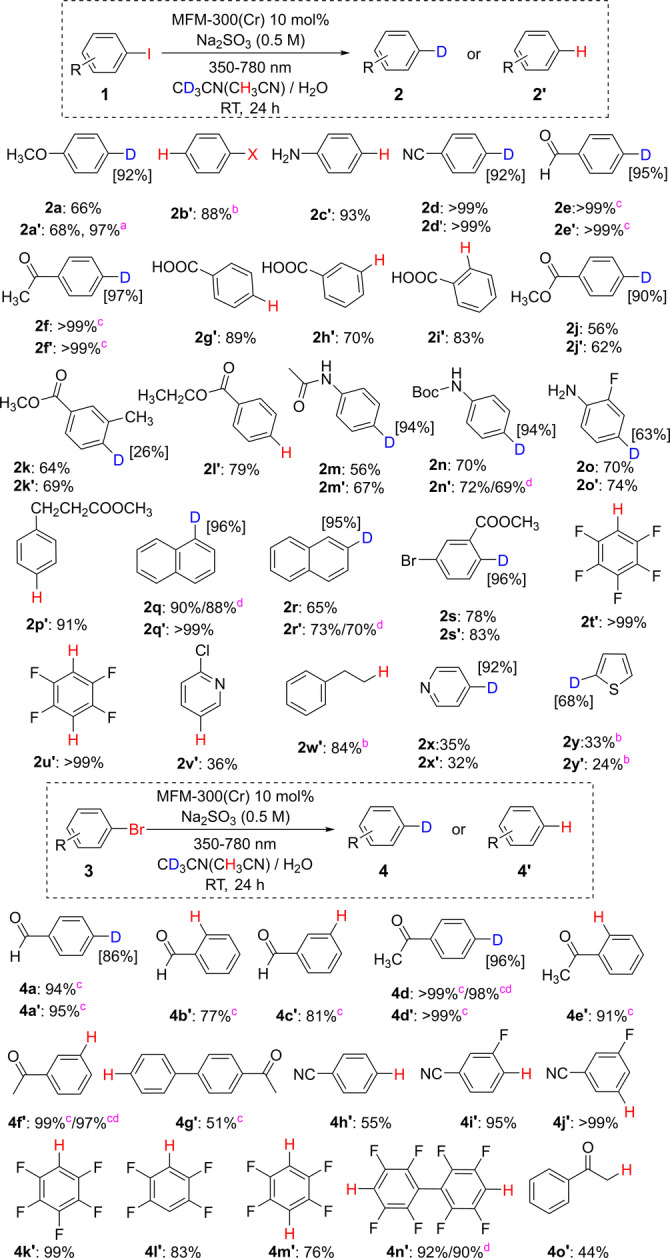
Photocatalytic dehalogenation of iodides and bromides over MFM‐300(Cr). Yields are given under the structure of each product and are determined by ^1^H NMR spectroscopy using nitromethane or cyclohexane as an internal standard. Several isolated yields (**2 n′**, **2 q**, **2 r′**, **4 d**, **4 f′**, **4 n′**) were also obtained. Full details are described in the Supporting Information. Typical reaction conditions: substrate (0.50 mmol), MFM‐300(Cr) (10 %, 0.05 mmol), CH_3_CN/H_2_O (15 mL/15 mL), Na_2_SO_3_ (0.5 M), 25 °C, 350–780 nm, 24 h. ^a^ 20 mol% of catalyst loading, ^b^ 48 h of reaction time, ^c^ in CH_2_Cl_2_/H_2_O or CD_2_Cl_2_/D_2_O solvent system, ^d^ isolated yield. The ^1^H and ^13^C NMR spectra of reaction mixtures of **2 a**, **2 a′**, **4 d** and **4 d′** are given in the Supporting Information.

The reactivity of a wide range of halogenated substrates was monitored under the above optimised conditions (Figure 3). Aryl, heteroaryl and alkyl halides show excellent yields of hydrodehalogenated (using CH_3_CN) and deuterodehalogenated (using CD_3_CN) products, with excellent functional group tolerance due to the mild reaction conditions applied. Importantly, 16 substrates show yields of >90 %. The substituents of iodobenzene (−COOH, **2 g**–**2 i**) and bromobenzene (−CHO and−COCH_3_, **4 a**–**4 f**) at *para*‐, *meta*‐, and *ortho*‐ positions give yields of 77–99 %, and these variations are ascribed to the effects of steric hindrance. The deuteration of halides containing carbonyl groups was more successful in CD_2_Cl_2_/H_2_O, which can suppress the side‐reaction of reductive coupling to form 1,2‐diols.^[33]^ Interestingly, substrates with different halogen substituents (F, Cl, Br) on the aryl rings show excellent chemoselectivity (iodides: **2 o**, **2 s**, **2 t**, **2 u**; bromides: **4 i**, **4 j**, **4 k**, **4 l**, **4 m** and **4 n**), attributed to the different bond dissociation energies and reduction potentials.[^39^, ^40^] Heteroaryl and alkyl halides have also been exploited (**2 v**, **2 w**, **2 x**, **2 y** and **4 o**), and broadens further the scope of this approach. Importantly, the photocatalytic efficiency is retained even with a 10 fold loading of the substrate (Figure S7), demonstrating the scalability of this method.

**Figure 4:**
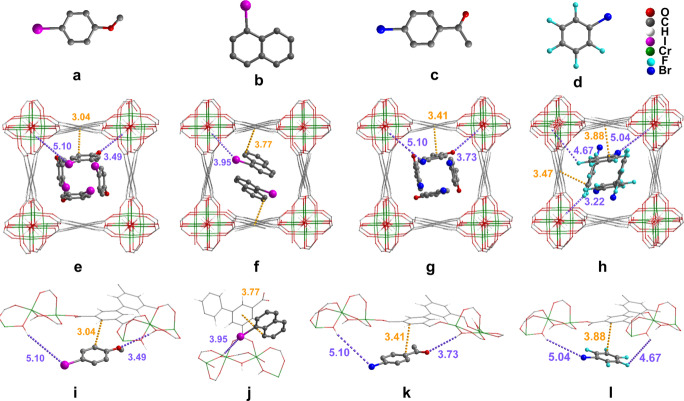
Views of crystal structures of MFM‐300(Cr) loaded with 4‐iodoanisole (I−PhOCH_3_), 1‐iodonaphthalene (I−Nap), 4′‐bromoacetophenone (Br−PhCOCH_3_) and bromopentafluorobenzene (Br−PhF_5_).^[44]^ All models were obtained from Rietveld refinements of SXPD data. The substrate molecules are disordered over two equally distributed sites in the pore and only one site is shown for clarity. (a–d) The chemical structures of 4‐iodoanisole, 1‐iodonaphthalene, 4′‐bromoacetophenone and bromopentafluorobenzene; (e–h) view of the structures of MFM‐300(Cr) ⋅ 0.920I‐PhOCH_3_ ⋅ 2H_2_O, MFM‐300(Cr) ⋅ 0.790I‐Nap ⋅ 5.486H_2_O, MFM‐300(Cr) ⋅ 1.036Br‐PhCOCH_3_ ⋅ 2H_2_O and MFM‐300(Cr) ⋅ 1.562Br‐PhF_5_ ⋅ 1.562H_2_O. (**i**–**l**) Detailed views of host–guest binding of adsorbed I−PhOCH_3_, I−Nap, Br−PhCOCH_3_ and Br−PhF_5_ molecules within MFM‐300(Cr). The guest molecules are highlighted by using amplified ball‐and‐stick model, hydrogen atoms are omitted for clarity.

To investigate the location of adsorbed substrates within the pores of MFM‐300(Cr), SXPD data were collected for MFM‐300(Cr) loaded with 4‐iodoanisole (I−PhOCH_3_), 1‐iodonaphthalene (I−Nap), 4′‐bromoacetophenone (Br−PhCOCH_3_) and bromopentafluorobenzene (Br−PhF_5_). Full structural analyses of the SXPD data have yielded highly satisfactory Rietveld refinements (Figures S8–S11, Tables S2–S6). As illustrated in Figure 4, the guest‐loaded MFM‐300(Cr) show full retention of the framework structure, and the substrate molecules are mainly stabilised by π⋅⋅⋅π interactions between the benzene rings of guest molecules and ligands of MFM‐300(Cr) with an inter‐planar distance of 3.04(3), 3.77(2), 3.41(2) and 3.88(1) Å for adsorbed I−PhOCH_3_, I−Nap, Br−PhCOCH_3_ and Br−PhF_5_, respectively. Additional hydrogen bonding between O or halogen atoms of guest molecules and the bridging −OH groups of MFM‐300(Cr) further stabilise the substrates within MFM‐300(Cr). Also, trace amounts of free water molecules were found in the structures, interacting with adsorbed guest substrates via additional hydrogen bonding. Thus, the SXPD studies reveal the confined binding of substrate molecules within the cavity of MFM‐300(Cr), and this has been confirmed by FTIR spectroscopic analyses (Figure S12 and SI). Host‐guest interactions can effectively promote the transfer of the photo‐induced electrons and activate the substrate for redox reactions, thus boosting the photocatalytic efficiency.

**Figure 5:**
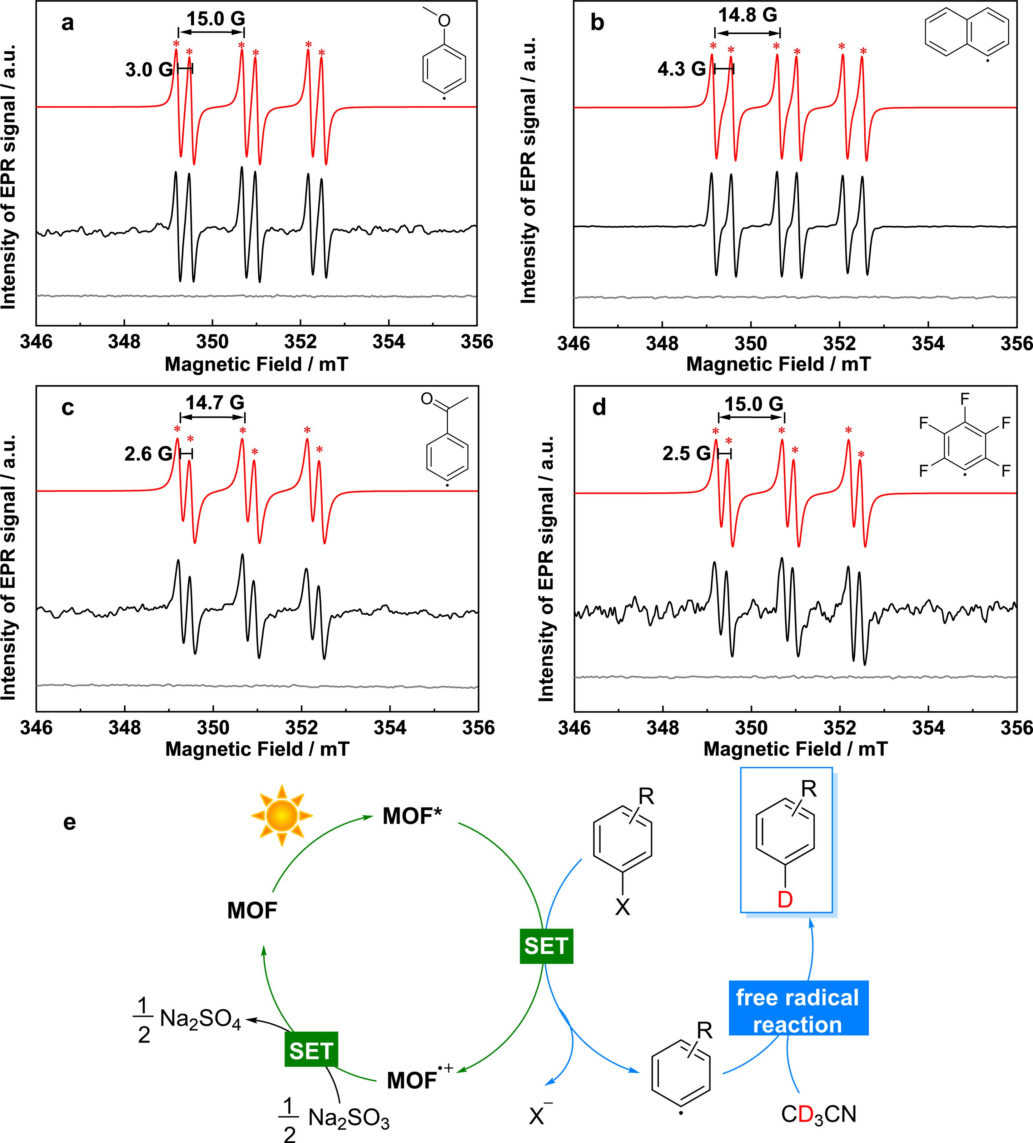
Mechanistic study. (a–d) X‐band EPR spectra of in situ photocatalytic reaction with different substrates (a) 4‐iodoanisole (I−PhOCH_3_), (b) 1‐iodonaphthalene (I−Nap), (c) 4′‐bromoacetophenone (Br−PhCOCH_3_) and (d) bromopentafluorobenzene (Br−PhF_5_) in the presence of the PBN spin trap. The structures of the carbon radicals that form the PBN adducts for each substrate are shown; (e) the proposed mechanism of photocatalytic dehalogenative deuteration of aryl halides over MFM‐300(Cr). SET=single electron transfer.

To investigate the reaction mechanism, in situ EPR spectroscopy was used to investigate any free radicals generated under photocatalytic conditions.^[41]^ α‐Phenyl N‐tertiary‐butyl nitrone (PBN), a nitrone spin‐trap known for trapping short lived radicals to generate a more stable free radical, was used as the spin trap to reactions with the four model substrates, I−PhOCH_3_, I−Nap, Br−PhCOCH_3_ and Br−PhF_5_, under optimised photocatalytic conditions. An intense six‐line signal characteristic of PBN‐radical adducts was found for each substrate, with subtly different hyperfine splitting parameters to the ^14^N and *β*‐^1^H of the nitroxide adduct formed (*a* ^*H*^ and *a* ^*N*^; Figure 5, Table S7). These parameters are consistent with C‐centred radicals, similar to those observed for PBN‐trapped Ph radicals,[^42^, ^43^] and assigned to the aryl‐based radicals PBN‐⋅PhOCH_3_, PBN‐⋅Nap, PBN‐⋅PhCOCH_3_ and PBN‐⋅PhF_5_, respectively. No radical was trapped or observed for reactions conducted without substrate (Figure S13) or under dark conditions (Figure 5). Thus, an aryl‐based radical has been identified as the reaction intermediate to the formation of dehalogenation products.

The following reaction mechanism is thus proposed (Figure 5d). Upon light irradiation, MFM‐300(Cr) is activated and photo‐induced electrons generated. These are transferred though single electron transfer (SET) to the adsorbed substrates via host–guest π⋅⋅⋅π interactions and hydrogen bonding, and the C−X bond of the substrate is cleaved to yield free aryl‐based radicals as confirmed by the EPR trapping experiments. The aryl‐based radicals can abstract a hydrogen or deuterium radical from the organic solvent molecules to afford the product. No ⋅CH_2_CN or ⋅CD_2_CN radicals were detected suggesting that the free radicals are rapidly scavenged. The hole left in MFM‐300(Cr) is reduced by Na_2_SO_3_, which is oxidised to Na_2_SO_4_, thus driving the electron‐hole separation and cycles of successive charge transfer reactions.

## Conclusion

MFM‐300(Cr) shows an excellent catalytic activity to promote the dehalogenative deuteration of a range of halides under mild conditions, representing the first example of MOF‐based photocatalyst for deuterium labelling of organic compounds. The photoinduced electrons have a high reduction potential (up to −2.55 V) that can reduce a wide range of substrates to their corresponding free radicals under mild conditions. The photocatalytic efficiency and selectively of MOFs can be tailored by the inherent flexibility in the design of MOFs, most notably for the control and refinement of host–guest interactions. Thus, using MOFs as photocatalysts for challenging organic reactions has significant prospects in medicinal and pharmaceutical chemistry.

## Conflict of interest

The authors declare no conflict of interest.

1

## Supporting information

As a service to our authors and readers, this journal provides supporting information supplied by the authors. Such materials are peer reviewed and may be re‐organized for online delivery, but are not copy‐edited or typeset. Technical support issues arising from supporting information (other than missing files) should be addressed to the authors.

Supporting Information

Supporting Information

Supporting Information

Supporting Information

Supporting Information

## Data Availability

The data that support the findings of this study are available from the corresponding author upon reasonable request.
