# Supplementary material for: Photocatalytic Dehalogenative Deuteration of Halides over a Robust Metal–Organic Framework
Source: Angew Chem Int Ed Engl. 2023 Oct 26;62(48):e202306267. doi: 10.1002/anie.202306267 (PMC10952292; doi:10.1002/anie.202306267)
Supplement: Supplementary file 5 — Supporting Information [file ANIE-62-0-s003.pdf]

Angewandte Chemie
Eine Zeitschrift der Gesellschaft Deutscher Chemiker

Supporting Information

Photocatalytic Dehalogenative Deuteration of Halides over a Robust Metal–Organic Framework

T. Luo, Z. Wang, Y. Chen, H. Li, M. Peng, F. Tuna, E. J. L. McInnes, S. J. Day, J. An, M. Schröder*, S. Yang**

Methods

Materials. $CrCl_3 \cdot 6H_2O$ (>98%) was purchased from Acros Organics. Sodium metabisulfite (reagent grade, $Na_2S_2O_5$ 97%), sodium sulfide hydrate ($Na_2S \cdot 3H_2O$, >60%), triethanolamine (TEOA, >99.0%) and hydrochloric acid (37%), acetonitrile (>99%) were purchased from Sigma-Aldrich Co., UK. N, N-dimethylformamide (DMF, >99%), sodium sulphite (Na_2SO_3, 98%), sodium hydroxide pearl (NaOH, >97%), dichloromethane (DCM), triethylamine (TEA, laboratory reagent grade) were bought from Fisher Chemical. Biphenyl-3,3',5,5'-tetracarboxylic acid (H_4L) was synthesised according to the literature procedure.[1] All chemicals and reagents used in this experiment were used as received without further purification.

Material characterisations.

PXRD data were collected on an X-ray powder diffractometer (Phillips X'pert MPD) with Cu-Kα radiation at a scan speed of 1.5 ^{o}/min.

TGA measurements were performed under air flow (5 mL/min) at a heating rate of 10 ^{o}C/min from 25 to 650 ^{o}C on a Pyris1 thermogravimetric analyser (Perkin-Elmer).

Elemental analysis for C, H and N content of MFM-300(Cr) was carried out using a CE-440 Elemental Analyser manufactured by Exeter Analytical. ICP-OES measurements for analysis of Cr were carried out using a Perkin-Elmer Optima 2000.

FTIR spectra were recorded using a Bruker Alpha II FT-IR spectrophotometer.

The recycled MFM-300(Cr) sample was characterised using the same above techniques as the fresh sample.

^{1}H NMR and ^{13}C NMR spectra were measured on Bruker B500 or B400 spectrometers.

GC-MS spectra were collected on 7890A series GC (Agilent) coupled to 5975C electron ionisation quadrupole mass spectrometer and 80 auto-sampler.

Synthesis of MFM-300(Cr). The synthesis of MFM-300(Cr) was conducted following our reported method.[2] $CrCl_3 \cdot 6H_2O$ (200 mg, 0.751 mmol) and H_4L (70.0 mg, 0.212 mmol) were added to a mixture of H_2O (10 mL) and HCl (1%, 1.5 mL). After being stirred at room temperature for 30 mins, the suspension was transferred into a 23 mL Teflon autoclave and heated at 210 °C for 3 days. After cooling to room temperature, the blue crystalline product was separated by centrifugation, washed with H_2O and hot DMF (3 x 20 mL) to remove unreacted metal salts and ligand. To remove residual DMF from the pores of MFM-300(Cr), soxhlet extraction was conducted with acetone for 2 days. After briefly drying in air, the obtained solid was activated under dynamic vacuum at 120 ^{o}C for 24 h.

General procedure for photocatalytic reaction. A 300 W Xe lamp (Zhongjiaojinyuan Co., Ltd) was used at a wavelength of 350-780 nm, and the distance between the lamp and quartz round bottom flask was fixed to 5.0 cm. Photocatalytic reactions were performed with a broad range of arylhalides and alkylkalides with electron-donating or electron-withdrawing substituents. In a typical photocatalytic reaction, substrate (0.500 mmol), desolvated MFM-300(Cr) (10 mol %) and Na_2SO_3 (1.89 g, 0.5 M) were added to a mixed solution of CH_3CN (or CD_3CN) with H_2O (15 mL/15 mL) in a quartz round bottom flask (50 mL). The above suspension

was sealed and sonicated for 30 sec to achieve uniform dispersion of the heterogeneous catalyst. After reaching the equilibrium at 25 °C with the aid of a water bath, the lamp was switched on to trigger the photoreaction. Upon completion, the catalyst MOF was separated by centrifugation, and the filtrate including the unreacted substrate and product was extracted using CH_2Cl_2 or ethyl acetate (3 x 15 mL). The filtrates were combined, the solvent removed by rotary evaporation and analysed by ^1H NMR spectroscopy using nitromethane or cyclohexane as internal standard in DMSO-d^6 or $CDCl_3$. For the recycling experiments, the recycled MOF catalyst was washed with acetone (3 x 10 mL) and dried in an oven overnight, and then activated under dynamic vacuum at 120 ºC for 24 h for additional recycling tests.

Structure determination and refinements of SPXRD data. SPXRD measurements were conducted at Beamline I11 Diamond Light Source (Oxford, UK) [λ = 0.826562(2) Å]. Desolvated MFM-300(Cr) was prepared by heating the as-synthesised sample at 150 ºC under vacuum for 1 day. To prepare the substrate-loaded samples, desolvated MOF (0.05 mmol) was dispersed in a 0.03 M of solution of the substrate 4-iodoanisole (I-$PhOCH_3$) in CH_3CN, 1-iodonaphthalene (I-Nap) in CH_3CN, 4'-bromoacetophenone (Br-$PhCOCH_3$) in CH_2Cl_2 and bromopentafluorobenzene (Br-PhF_5) in CH_3CN. After being soaked for 2 days, the MOF was filtered, washed with the corresponding solvent and dried. The powder sample was loaded in a 0.7 mm borosilicate glass capillary to prevent preferred orientations. High-resolution synchrotron PXRD data were collected in the 2θ range of 0 - 150° with a step size of 0.001° using multi-analyser crystal (MAC) detectors at 25.0 °C.

TOPAS 5 was used to perform Pawley and Rietveld refinement on the PXRD patterns.[3] Background, cell parameters and peak profile with Stephen model[4] were first refined using Pawley refinement and then transferred to Rietveld refinement. The scale factor and lattice parameters were allowed to refine for all the diffraction patterns. The refined structural parameters include the fractional coordinates (x, y, z), the isotropic displacement factors for all the atoms, and the site occupancy factors (SOF) for the framework and guest molecules. The final stage of Rietveld refinement involved soft restraints to the C–C bond lengths within the benzene rings, and rigid body refinement was applied to the guest molecules in the pore. The quality of the Rietveld refinements was confirmed by the low weighted profile factors and the good fit to the data with reasonable isotropic displacement factors within experimental error (Tables S2–S6).

EPR measurements. Continuous wave EPR measurements were carried out at X-band (9.85 GHz) using an EMX Micro spectrometer (Bruker). Modulation amplitude of 0.9 mT was used with a microwave power of ~ 2.0 mW based on spectral lines saturation test. Strong pitch (g = 2.0028) was used as a standard reference. Theoretical modelling of EPR spectra was performed using the Easyspin toolbox package (Version 6.0.0) in MATLAB software (version R2020a).[5]

All reagents were deoxygenated under Ar. For the *in situ* EPR measurements, I-$PhOCH_3$, I-Nap, Br-$PhCOCH_3$ and Br-PhF_5 were chosen as model substrates. In a typical experiment, α-phenyl N-tertiary-butyl nitrone (PBN) was dissolved in CH_3CN (0.2 mol/L) and used as a spin trap. Substrates (0.05 mmol), MFM-300(Cr) (10 mol%, 0.005 mmol), and Na_2SO_3 (0.5 M) were dissolved in a mixed solution of CH_3CN/H_2O (1.5

mL/1.5 mL) in a deoxygenated vial under Ar, followed by 0.1 mL of the PBN stock solution. CH_3CN was replaced by CH_2Cl_2 when Br-$PhCOCH_3$ was used as substrate. 0.5 mL of the resultant PBN mixed solution was then transferred into a capillary for freeze pumping to further degas the solution in order to fully remove all the dissolved gases (<0.01 mbar). The capillary was then flame-sealed and directly used for EPR measurements. *In situ* EPR spectra of the photocatalytic reactions were collected before (dark) and after (light) radiation (350-780 nm) by a Xe lump for 10 mins.

Figures

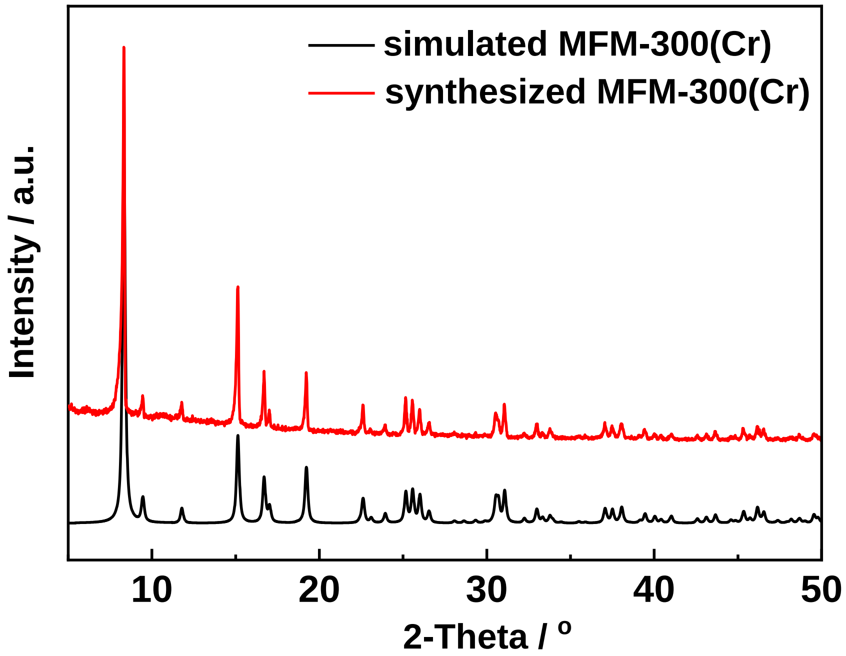

Fig. S1 PXRD patterns of simulated and as-synthesised MFM-300(Cr).

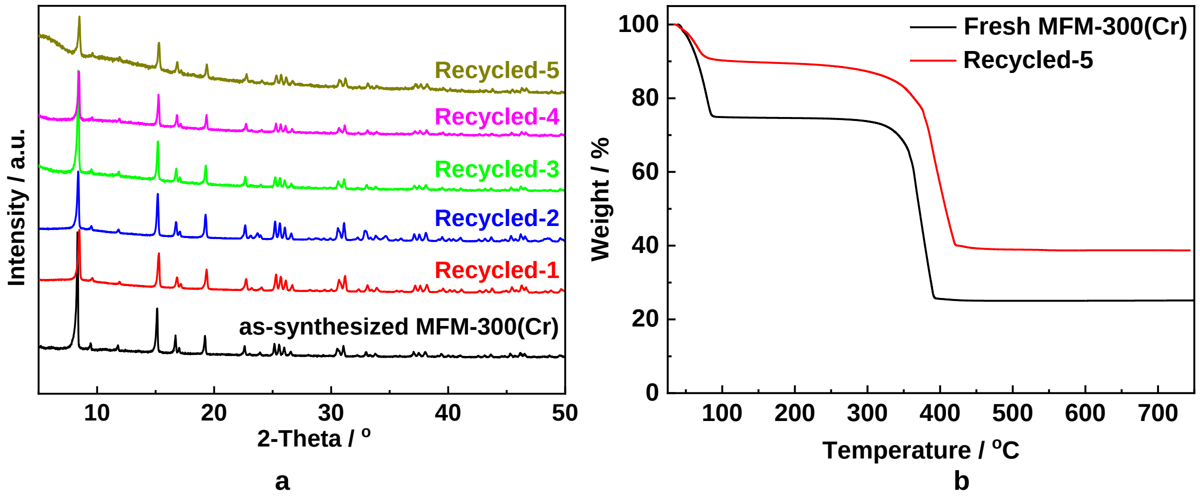

Fig. S2 PXRD patterns of fresh and recycled samples of MFM-300(Cr). Photocatalytic reaction conditions: 4-iodoanisole (0.500 mmol), MFM-300(Cr) (10 mol%, 0.05 mmol), CH_3CN/H_2O (15 mL/15 mL), Na_2SO_3 (0.5 M), 25 ºC, 350-780 nm, light irradiation for 24 h.

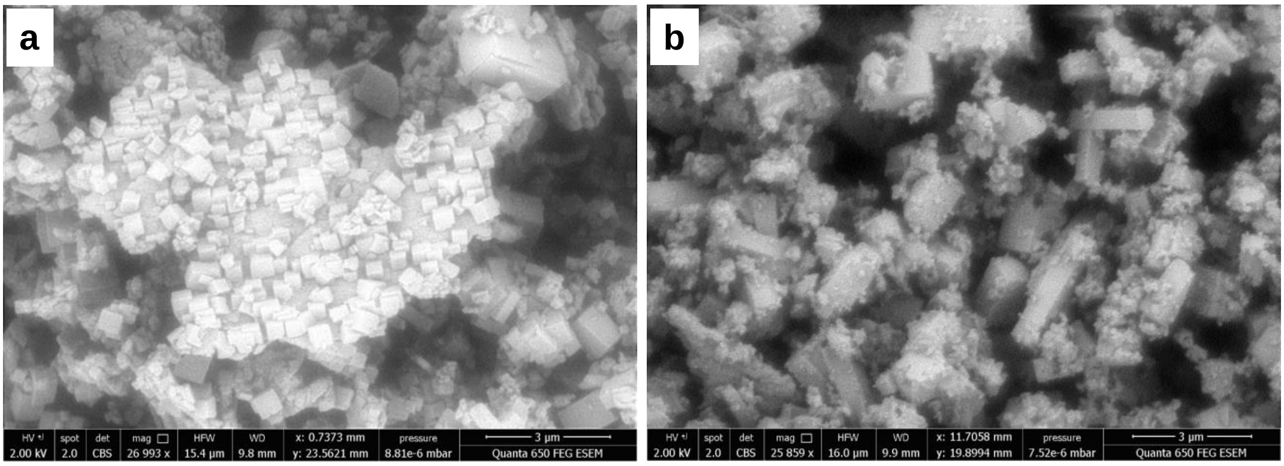

Fig. S3 SEM images of fresh MFM-300(Cr) (a) and recycled sample after 5 cycles of photocatalytic reactions (b).

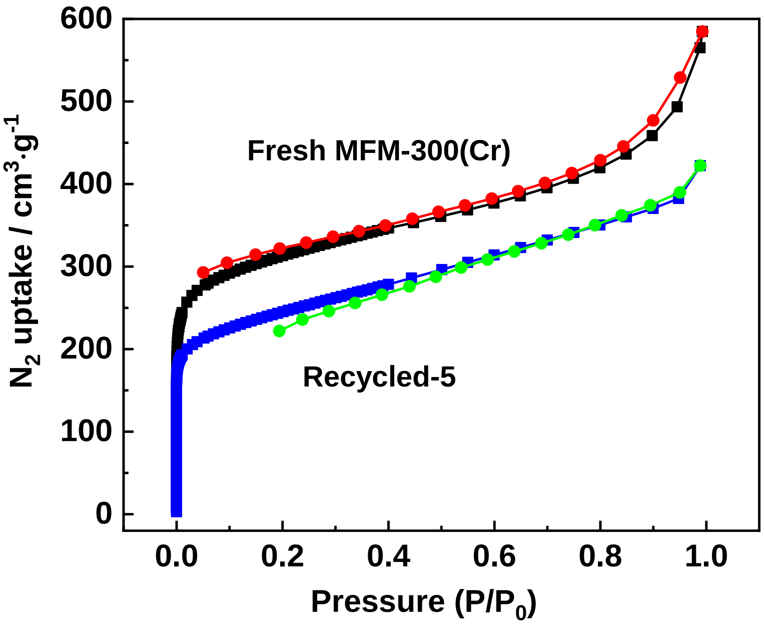

Fig. S4 N_2 adsorption/desorption isotherm of desolvated MFM-300(Cr) and recycled MOF sample after 5 cycles of photocatalytic reactions (Recyled-5) at 77 K. The BET surface area is 1147 and 903 m^2/g for the fresh and used catalyst, respectively.

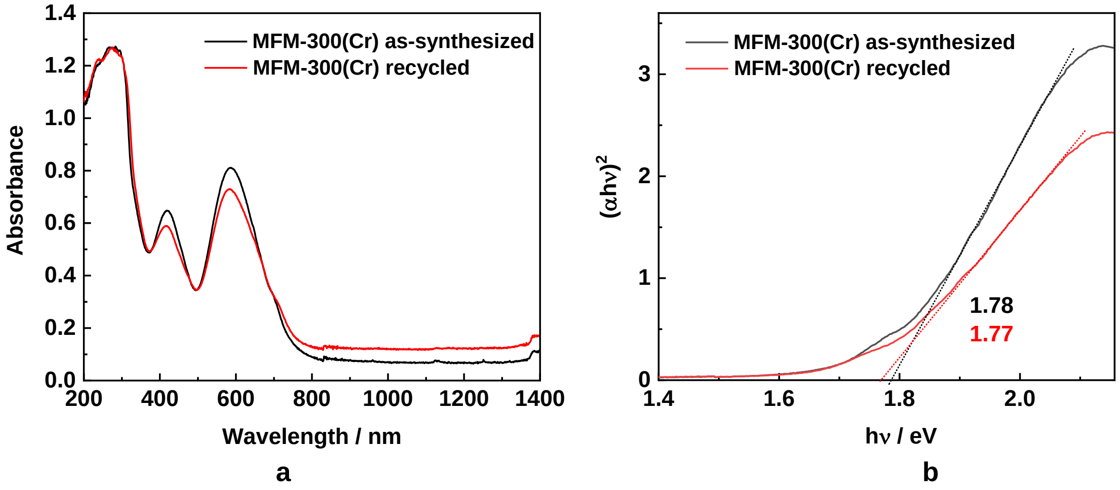

Fig. S5 (a) UV/Vis diffuse reflectance spectrum (DRS) spectrum of fresh and recycled MFM-300(Cr) (Recycled-5), and (b) the tauc-plot for the bandgap calculation. The band gaps were calculated as 1.78 and 1.77 eV for fresh and recycled MFM-300(Cr), respectively.

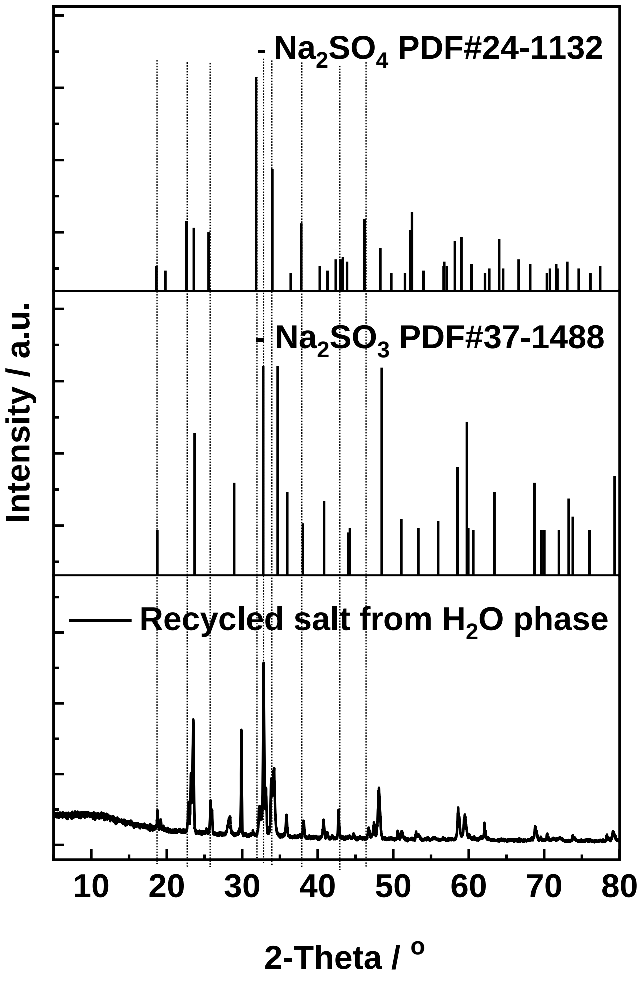

Fig. S6 PXRD patterns of the recycled inorganic salt from the H_2O phase.

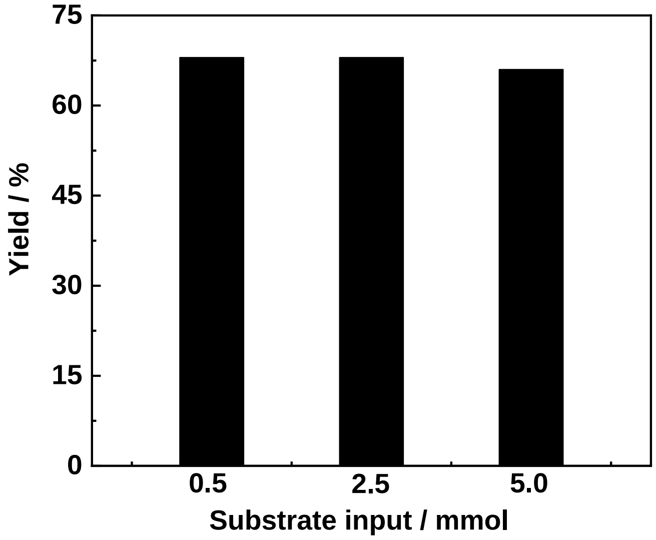

Fig. S7 Scale-up experiments for photocatalytic dehalogenation of 4-iodoanisole over MFM-300(Cr). Photocatalytic reaction conditions: 4-iodoanisole (0.500 mmol = 0.117 g / 2.50 mmol = 0.585 g / 5.00 mmol = 1.17 g), MFM-300(Cr) (10 mol%, 0.05 mmol / 0.25 mmol / 0.5 mmol), CH_3CN/H_2O (15 mL/15 mL, 75 mL/75 mL, 150 mL/150 mL), Na_2SO_3 (0.5 M), 350-780 nm, light irradiation for 24 h.

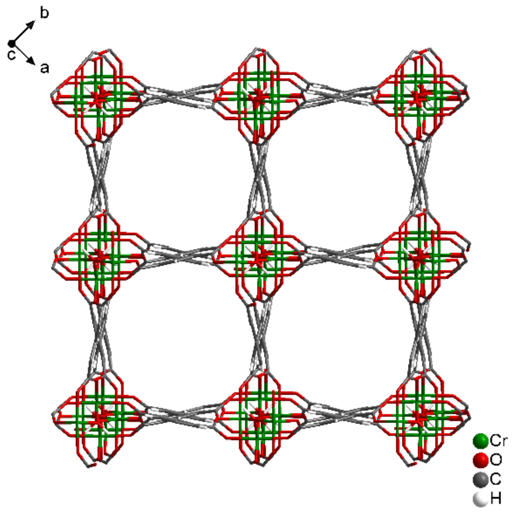

Fig. S8 View of the crystal structure of MFM-300(Cr) along the *c* axis showing pore size of ~7.5 Å.

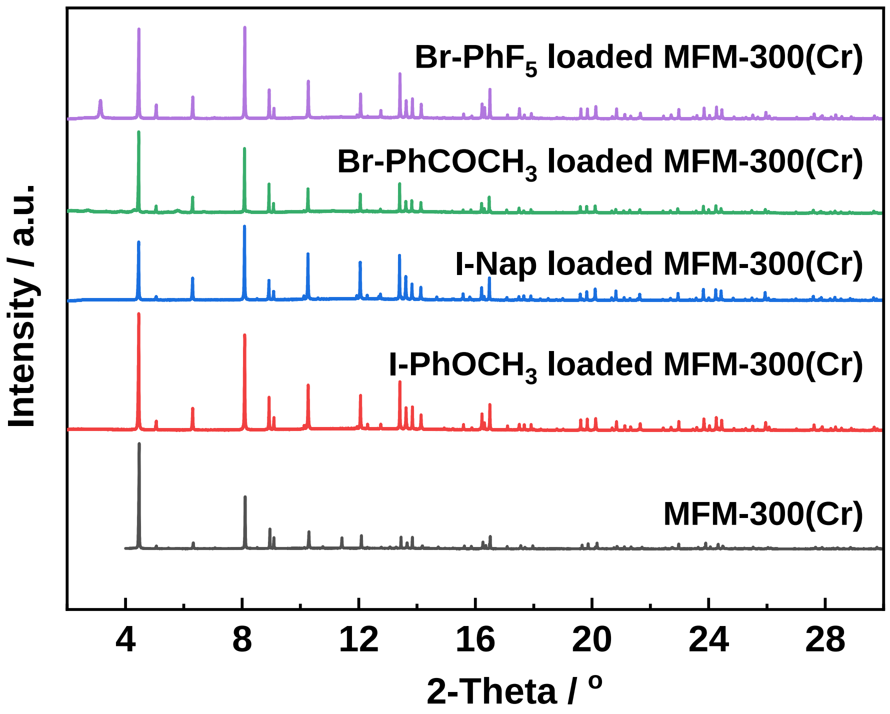

Fig. S9 SXPD patterns of as-synthesized and guest-loaded MFM-300(Cr).

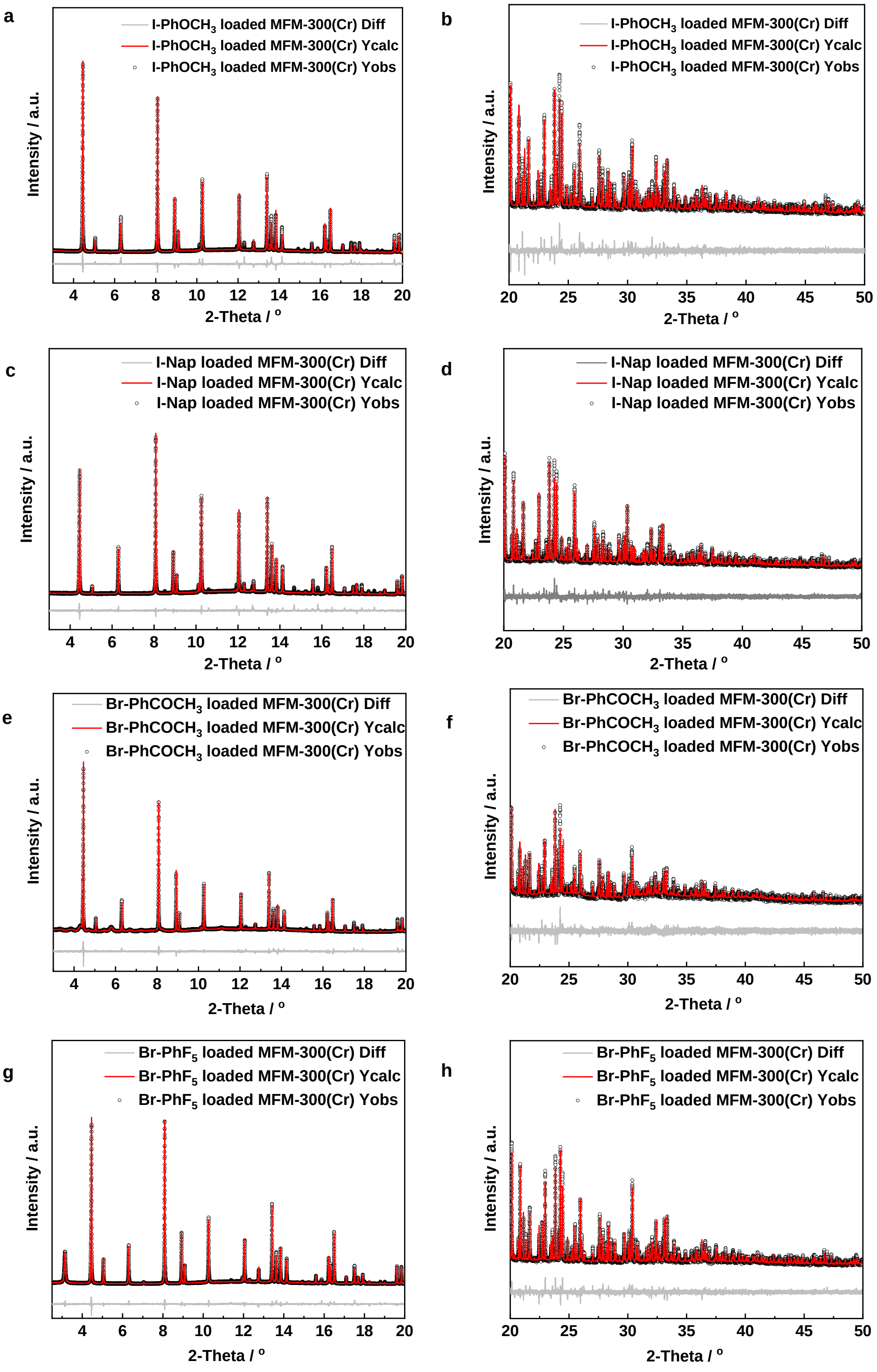

Fig. S10 Experimental data (black), Rietveld refinement (red) and the difference between them (grey) for SXPD patterns of (a,b) I-PhOCH$_3$-loaded MFM-300(Cr); (c,d) I-Nap-loaded MFM-300(Cr); (e,f) Br-PhCOCH$_3$-loaded MFM-300(Cr); (g,h) Br-PhF$_5$-loaded MFM-300(Cr) at room temperature [λ = 0.826562(2) Å].

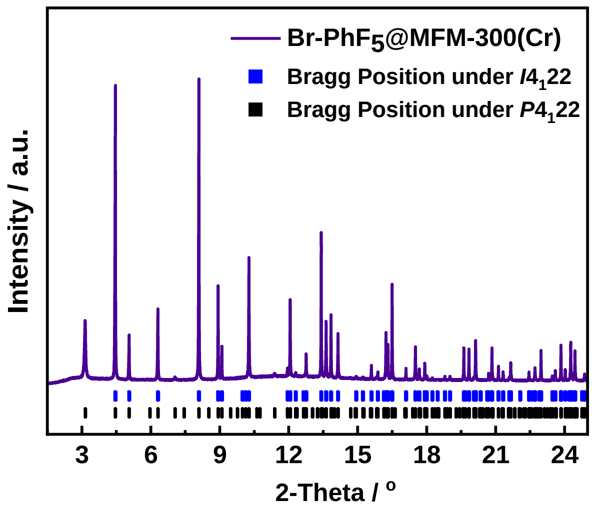

Fig. S11 Experimental data of Br-PhF_5-loaded MFM-300(Cr) and the Bragg peak position under symmetry of $I4_122$ (blue) and $P4_122$ (black).

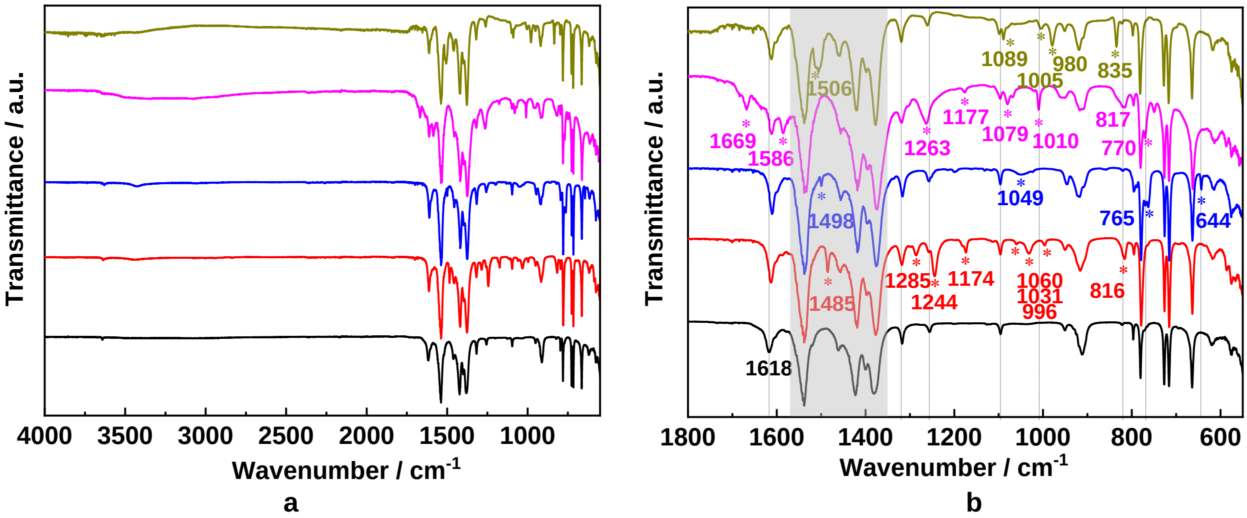

Fig. S12 FT-IR spectra of as-synthesised MFM-300(Cr) (black), I-$PhOCH_3$-loaded MFM-300(Cr) (red), I-Nap-loaded MFM-300(Cr) (blue), Br-$PhCOCH_3$-loaded MFM-300(Cr) (pink) and Br-PhF_5-loaded MFM-300(Cr) (green). (a) Full spectra, and (b) magnified spectra in the region 1800-550 cm^{-1}.

The adsorption of substrates (I-$PhOCH_3$, I-Nap, Br-$PhCOCH_3$ and Br-PhF_5) in MFM-300(Cr) and the formation of host-guest binding interactions have been confirmed by FTIR spectra. Bands in the range 1540-1380 cm^{-1} and 850-650 cm^{-1} are assigned to the skeletal vibration of benzene rings and the bending modes of aromatic C-H groups of the organic ligands, respectively.[6] Upon adsorption of the guest molecules, new peaks and small peak shifts are observed in these two band ranges, indicating host-guest interactions between the guest molecules and MOF frameworks. Moreover, a range of new peaks marked with asterisks in Fig. S13 demonstrate that the substrate molecules have been successfully loaded. For example, the spectrum of Br-$PhCOCH_3$-loaded MFM-300(Cr) (pink) shows a new peak of 1669 cm^{-1} assigned to the υ(C=O) stretching vibration of Br-$PhCOCH_3$.[7] The peak originally at 1618 cm^{-1} is split into two peaks, 1614 and 1588 cm^{-1},

ascribed to hydrogen bonding between the –OH groups of MFM-300(Cr) and the carbonyl groups of the substrates.[8]

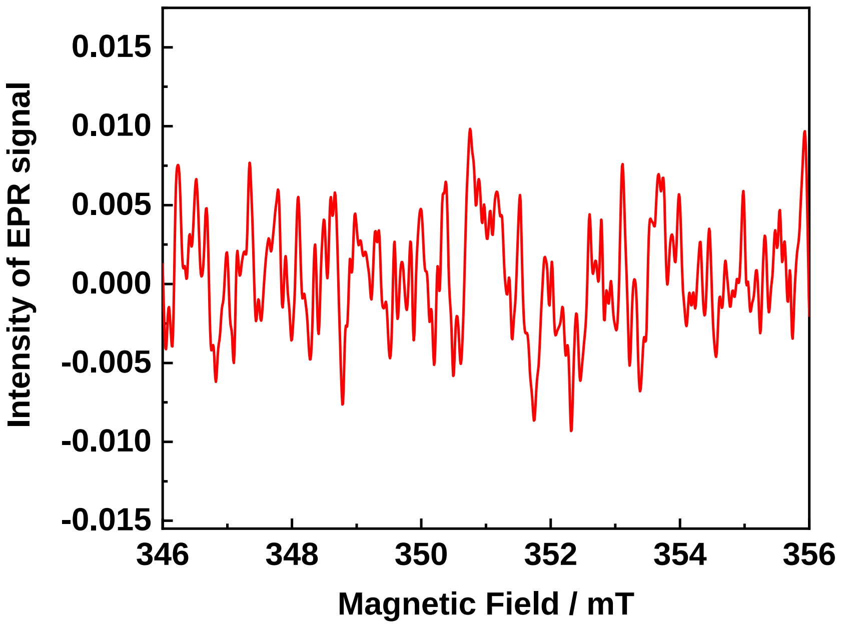

Fig. S13 ***In situ*** **EPR spectrum of photocatalytic reaction without adding substrate.** Conditions: MFM-300(Cr) (10%, 0.005 mmol), Na_2SO_3 (0.5 M), PBN (0.2 mol/L), CH_3CN/H_2O (1.5 mL/1.5 mL), 350-780 nm irradiation for 10 minutes.

Tables

Table S1 Summary of catalysis results for control experiments.

Entry	Catalyst	Reducing agent	Wavelength / nm	Solvents	X/H Yield / %
1	MFM-300(Cr)/20 mol%	Na_2SO_3	350-780	CH_3CN/H_2O	97%
2	MFM-300(Cr)	Na_2SO_3	350-780	CH_3CN/H_2O	68%
3	MFM-300(Cr)	Na_2SO_3/0.25 M	350-780	CH_3CN/H_2O	48%
4	MFM-300(Cr)	Na_2SO_3	350-780	CH_3CN	12%
5	MFM-300(Cr)	Na_2SO_3	350-780	DCM/H_2O	52%
6	n.a.	n.a.	350-780	CH_3CN/H_2O	4%
7	MFM-300(Cr)	n.a.	350-780	CH_3CN/H_2O	7%
8	n.a.	Na_2SO_3	350-780	CH_3CN/H_2O	34%
9	MFM-300(Cr)	Na_2SO_3	dark	CH_3CN/H_2O	0%
10	$CrCl_3\cdot 6H_2O$-H_4L	Na_2SO_3	350-780	CH_3CN/H_2O	34%

Typical reaction conditions: 4-iodoanisole (0.50 mmol), MFM-300(Cr) (10 mol%, 0.05 mmol), CH_3CN/H_2O (15 mL/15 mL), Na_2SO_3 (0.5 M), 25 ºC, 350-780 nm, 24 h. For entry 3 and 6, 30 mL of organic solvent was added; for entry 4, 15mL of CH_2Cl_2 was added; for entry 10, a powdered mixture of $CrCl_3\cdot 6H_2O$ (0.1 mmol, 0.027g) and H_4L (0.05 mmol, 0.0165g) was used (H_4L = biphenyl-3, 3', 5, 5'-tetracarboxylic acid).

Table S2 Crystallographic data and details of bare and substrate-loaded MFM-300(Cr).

Sample	I-$PhOCH_3$-loaded MFM-300(Cr)	I-Nap-loaded MFM-300(Cr)	Br-$PhCOCH_3$-loaded MFM-300(Cr)	Br-PhF_5-loaded MFM-300(Cr)
Crystal system	Tetragonal	Tetragonal	Tetragonal	Tetragonal
Space group	$I4_122$	$I4_122$	$I4_122$	$P4_122$
Chemical formula	$CrC_8O_5H_4\cdot 0.460C_7H_7OI\cdot H_2O$	$CrC_8O_5H_4\cdot 0.395C_{10}H_7I\cdot 2.743H_2O$	$CrC_8O_5H_4\cdot 0.518C_8H_7OBr\cdot H_2O$	$CrC_8O_5H_4\cdot 0.545C_6F_5Br\cdot 0.781H_2O$
2θ range for refinement	3-50°	3-50°	3-50°	2.5-50°
Refinement methods	Rietveld Method	Rietveld Method	Rietveld Method	Rietveld Method
a (Å)	15.01843(9)	15.02952(9)	15.02552(10)	15.01298(7)
c (Å)	11.96891(7)	11.98429(8)	11.99265(8)	11.97045(6)
V (Å^3)	2699.63(3)	2707.09(4)	2707.53(4)	2698.02(3)
R_{wp} (%)	7.929	7.199	6.545	6.894
R_p(%)	5.688	5.139	5.047	5.153
R_{Bragg}(%)	4.791	2.344	3.256	2.750
R_{exp} (%)	3.741	4.002	3.948	4.063
Gof χ^2	2.120	1.799	1.658	1.697

Table S3 Atomic parameters from the Rietveld refinement of I-$PhOCH_3$-loaded MFM-300(Cr).

Species	Atom	*x*	*y*	*z*	SOF	B_{eq} (Å^2)	Wyckoff
MFM-300(Cr)	Cr	0.69110(5)	0.30890(5)	0.5	1	0	8*e*
	O1	0.7511(2)	0.25	0.625	1	0.50(4)	8*f*
	O2	0.6193(5)	0.3789(5)	0.6069(4)	1	0.50(4)	16*g*
	O3	0.6064(5)	0.2898(4)	0.7485(5)	1	0.50(4)	16*g*
	C1	0.5942(4)	0.3605(3)	0.7010(4)	1	1.12(6)	16*g*
	C2	0.54404(15)	0.43097(13)	0.7636(3)	1	1.12(6)	16*g*
	C3	0.5	0.5	0.7042(4)	1	1.12(6)	8*c*
	C4	0.54404(15)	0.43097(13)	0.8822(2)	1	1.12(6)	16*g*
	C5	0.5	0.5	0.9415(2)	1	1.12(6)	8*c*
	H1	0.823(3)	0.25	0.625	1	0.61(5)	8*f*
	H3	0.5	0.5	0.6165(17)	1	1.34(7)	8*c*
	H4	0.5766(6)	0.3799(10)	0.9261(8)	1	1.34(7)	16*g*
Water	OW1	-0.0059(4)	0.25	0.625	1	12.3(4)	8*f*
I-$PhOCH_3$	C_1	0.1588(15)	0.3355(10)	0.2898(6)	0.2299(6)	15.00(17)	16*g*
	C_2	0.222(3)	0.4031(19)	0.2882(11)	0.2299(6)	15.00(17)	16*g*
	C_3	0.242(3)	0.446(3)	0.1888(14)	0.2299(6)	15.00(17)	16*g*
	C_4	0.199(3)	0.422(2)	0.0901(12)	0.2299(6)	15.00(17)	16*g*
	C_5	0.135(2)	0.3540(18)	0.0922(7)	0.2299(6)	15.00(17)	16*g*
	C_6	0.1154(15)	0.3111(13)	0.1924(5)	0.2299(6)	15.00(17)	16*g*
	C_7	0.244(7)	0.419(4)	-0.101(2)	0.2299(6)	15.00(17)	16*g*
	O_1	0.223(4)	0.468(3)	-0.0030(15)	0.2299(6)	15.00(17)	16*g*
	I_1	0.1289(2)	0.2701(2)	0.4413(3)	0.2299(6)	15.00(17)	16*g*
	H_1	0.256(3)	0.422(2)	0.3640(12)	0.2299(6)	18.0(2)	16*g*
	H_2	0.291(4)	0.498(3)	0.1859(18)	0.2299(6)	18.0(2)	16*g*
	H_3	0.100(2)	0.334(2)	0.0173(7)	0.2299(6)	18.0(2)	16*g*
	H_4	0.0660(12)	0.2586(15)	0.1932(5)	0.2299(6)	18.0(2)	16*g*
	H_5	0.189(11)	0.377(15)	-0.128(14)	0.2299(6)	18.0(2)	16*g*
	H_6	0.302(15)	0.375(15)	-0.087(9)	0.2299(6)	18.0(2)	16*g*
	H_7	0.26(2)	0.463(4)	-0.170(7)	0.2299(6)	18.0(2)	16*g*

Table S4 Atomic parameters from the Rietveld refinement of I-Nap-loaded MFM-300(Cr).

Species	Atom	*x*	*y*	*z*	SOF	B_{eq} (Å^2)	Wyckoff
MFM-300(Cr)	Cr	0.69143(5)	0.30857(5)	0.5	1	0.61(2)	8*e*
	O1	0.7481(2)	0.25	0.625	1	0.50(4)	8*f*
	O2	0.6194(4)	0.3813(5)	0.6049(4)	1	0.50(4)	16*g*
	O3	0.5996(5)	0.2866(3)	0.7478(4)	1	0.50(4)	16*g*
	C1	0.5912(3)	0.3596(3)	0.6990(3)	1	0.88(5)	16*g*
	C2	0.54102(12)	0.43001(10)	0.7614(2)	1	0.88(5)	16*g*
	C3	0.5	0.5	0.7027(3)	1	0.88(5)	8*c*
	C4	0.54102(12)	0.43001(10)	0.87890(18)	1	0.88(5)	16*g*
	C5	0.5	0.5	0.93763(16)	1	0.88(5)	8*c*
	H1	0.808(3)	0.25	0.625	1	0.60(5)	8*f*
	H3	0.5	0.5	0.6151(16)	1	1.06(7)	8*c*
	H4	0.5716(6)	0.3778(10)	0.9227(8)	1	1.06(7)	16*g*
Water	OW1	-0.0487(9)	0.25	0.625	0.743(12)	13.1(6)	8*f*
	OW2	0.2636(4)	0.3989(5)	0.6384(6)	1.000(11)	15.2(4)	16*g*
I-Nap	C_1	0.1308(7)	0.2395(13)	0.0545(12)	0.1974(9)	10.00(15)	16*g*
	C_2	0.1426(9)	0.1622(13)	-0.0041(13)	0.1974(9)	10.00(15)	16*g*
	C_3	0.1770(12)	0.0864(13)	0.0500(15)	0.1974(9)	10.00(15)	16*g*
	C_4	0.1988(11)	0.0893(14)	0.1610(15)	0.1974(9)	10.00(15)	16*g*
	C_5	0.1875(8)	0.1680(14)	0.2242(13)	0.1974(9)	10.00(15)	16*g*
	C_6	0.2085(10)	0.1762(15)	0.3395(13)	0.1974(9)	10.00(15)	16*g*
	C_7	0.1960(13)	0.2544(16)	0.3963(13)	0.1974(9)	10.00(15)	16*g*
	C_8	0.1615(15)	0.3295(16)	0.3406(13)	0.1974(9)	10.00(15)	16*g*
	C_9	0.1403(12)	0.3249(14)	0.2298(13)	0.1974(9)	10.00(15)	16*g*
	C_10	0.1525(8)	0.2451(13)	0.1692(12)	0.1974(9)	10.00(15)	16*g*
	H_1	0.1044(8)	0.2982(13)	0.0137(13)	0.1974(9)	12.00(18)	16*g*
	H_2	0.1257(11)	0.1591(14)	-0.0918(13)	0.1974(9)	12.00(18)	16*g*
	H_3	0.1863(16)	0.0253(14)	0.0035(17)	0.1974(9)	12.00(18)	16*g*
	H_4	0.2251(13)	0.0307(14)	0.2016(17)	0.1974(9)	12.00(18)	16*g*
	H_7	0.2125(15)	0.2589(18)	0.4839(13)	0.1974(9)	12.00(18)	16*g*
	H_8	0.1521(19)	0.3907(17)	0.3868(14)	0.1974(9)	12.00(18)	16*g*
	H_9	0.1138(14)	0.3825(14)	0.1867(14)	0.1974(9)	12.00(18)	16*g*
	I_1	0.2606(13)	0.0670(17)	0.4304(17)	0.1974(9)	10.00(15)	16*g*

Table S5 Atomic parameters from the Rietveld refinement of Br-$PhCOCH_3$-loaded MFM-300(Cr).

Species	Atom	*x*	*y*	*z*	SOF	B_{eq} (Å^2)	Wyckoff
MFM-300(Cr)	Cr	0.69053(7)	0.30947(7)	0.5	1	0	8*e*
	O1	0.7498(3)	0.25	0.625	1	0.76(6)	8*f*
	O2	0.6200(6)	0.3779(7)	0.6061(5)	1	0.76(6)	16*g*
	O3	0.6053(7)	0.2880(4)	0.7474(6)	1	0.76(6)	16*g*
	C1	0.5935(5)	0.3601(4)	0.7008(4)	1	0.50(8)	16*g*
	C2	0.54365(18)	0.43077(15)	0.7633(4)	1	0.50(8)	16*g*
	C3	0.5	0.5	0.7041(5)	1	0.50(8)	8*c*
	C4	0.54365(18)	0.43077(15)	0.8817(3)	1	0.50(8)	16*g*
	C5	0.5	0.5	0.9409(3)	1	0.50(8)	8*c*
	H1	0.810(4)	0.25	0.625	1	0.91(7)	8*f*
	H3	0.5	0.5	0.617(2)	1	0.60(9)	8*c*
	H4	0.5759(8)	0.3796(12)	0.9254(10)	1	0.60(9)	16*g*
Water	OW1	-0.0410(7)	0.25	0.625	0.999(13)	17.9(7)	8*f*
Br-$PhCOCH_3$	C_1	0.1434(6)	0.3023(5)	0.3176(4)	0.2588(7)	10.0(2)	16*g*
	C_2	0.1956(12)	0.3767(9)	0.2956(6)	0.2588(7)	10.0(2)	16*g*
	C_3	0.2083(16)	0.4028(12)	0.1861(7)	0.2588(7)	10.0(2)	16*g*
	C_4	0.1696(14)	0.3555(11)	0.0983(5)	0.2588(7)	10.0(2)	16*g*
	C_5	0.1178(10)	0.2808(10)	0.1226(4)	0.2588(7)	10.0(2)	16*g*
	C_6	0.1044(7)	0.2539(7)	0.2321(4)	0.2588(7)	10.0(2)	16*g*
	C_7	0.1851(18)	0.3873(15)	-0.0183(7)	0.2588(7)	10.0(2)	16*g*
	C_8	0.1431(18)	0.3363(16)	-0.1136(5)	0.2588(7)	10.0(2)	16*g*
	O_1	0.231(2)	0.4526(17)	-0.0360(10)	0.2588(7)	10.0(2)	16*g*
	Br_1	0.1249(3)	0.2665(3)	0.4682(4)	0.2588(7)	10.0(2)	16*g*
	H_1	0.2256(15)	0.4135(10)	0.3634(8)	0.2588(7)	12.0(2)	16*g*
	H_2	0.248(2)	0.4604(15)	0.1664(10)	0.2588(7)	12.0(2)	16*g*
	H_3	0.0871(12)	0.2429(12)	0.0564(4)	0.2588(7)	12.0(2)	16*g*
	H_4	0.0642(9)	0.1961(8)	0.2507(5)	0.2588(7)	12.0(2)	16*g*
	H_5	0.0707(18)	0.3349(16)	-0.1053(5)	0.2588(7)	12.0(2)	16*g*
	H_6	0.1662(16)	0.2674(17)	-0.1145(4)	0.2588(7)	12.0(2)	16*g*
	H_7	0.161(2)	0.3684(18)	-0.1916(6)	0.2588(7)	12.0(2)	16*g*

Table S6 Atomic parameters from the Rietveld refinement of Br-PhF_5-loaded MFM-300(Cr).

Species	Atom	*x*	*y*	*z*	SOF	B_{eq} (Å^2)	Wyckoff
MFM-300(Cr)	Cr1	0.05801(11)	0.94199(11)	0.125	1	0.642(19)	4*e*
	Cr2	0.44249(11)	0.55751(11)	0.125	1	0.642(19)	4*e*
	O1	0.4883(5)	0.5	0.25	1	0.50(3)	4*e*
	O1B	-0.0041(4)	1	0.25	1	0.50(3)	4*e*
	H1	0.548(2)	0.5	0.25	1	0.60(4)	4*e*
	H1B	-0.064(2)	1	0.25	1	0.60(4)	4*e*
	C1	0.3461(2)	0.6080(2)	0.3291(3)	1	0.95(4)	8*f*
	C1B	0.1573(2)	0.8899(2)	0.3290(3)	1	0.95(4)	8*f*
	C2	0.29454(6)	0.68177(7)	0.3895(2)	1	0.95(4)	8*f*
	C2B	0.20546(6)	0.81823(7)	0.3895(2)	1	0.95(4)	8*f*
	C3	0.25	0.75	0.3305(3)	1	0.95(4)	8*f*
	C4	0.29454(6)	0.68177(7)	0.50752(17)	1	0.95(4)	8*f*
	C4B	0.20546(6)	0.81823(7)	0.50752(17)	1	0.95(4)	8*f*
	C5	0.25	0.75	0.56652(16)	1	0.95(4)	8*f*
	H3	0.25	0.75	0.2546(13)	1	1.14(5)	8*f*
	H4	0.3232(5)	0.6379(7)	0.5455(6)	1	1.14(5)	8*f*
	H4B	0.1768(5)	0.8621(7)	0.5455(6)	1	1.14(5)	8*f*
	O2	0.3759(4)	0.6290(3)	0.2357(3)	1	0.50(3)	8*f*
	O2B	0.1503(4)	0.9614(3)	0.3809(4)	1	0.50(3)	8*f*
	O3	0.3517(4)	0.5364(3)	0.3811(4)	1	0.50(3)	8*f*
	O3B	0.1301(4)	0.8690(3)	0.2343(3)	1	0.50(3)	8*f*
Water	OW1	0.7024(12)	0.5	0.25	0.953(16)	20.0(10)	4*f*
	Ow_1B	0.8120(10)	1	0.25	0.608(13)	5.1(6)	4*f*
Br-PhF_5	C_1	0.5278(4)	-0.1209(3)	-1.1225(5)	0.2872(19)	10.00(18)	8*g*
	C_2	0.4844(4)	-0.1392(4)	-1.2223(5)	0.2872(19)	10.00(18)	8*g*
	C_3	0.5154(5)	-0.1022(5)	-1.3212(5)	0.2872(19)	10.00(18)	8*g*
	C_4	0.5899(5)	-0.0474(5)	-1.3201(5)	0.2872(19)	10.00(18)	8*g*
	C_5	0.6329(5)	-0.0296(6)	-1.2199(6)	0.2872(19)	10.00(18)	8*g*
	C_6	0.6023(4)	-0.0660(5)	-1.1202(5)	0.2872(19)	10.00(18)	8*g*
	F_1	0.4130(5)	-0.1919(6)	-1.2234(6)	0.2872(19)	10.00(18)	8*g*
	F_2	0.4739(6)	-0.1192(7)	-1.4170(5)	0.2872(19)	10.00(18)	8*g*
	F_3	0.6197(7)	-0.0122(7)	-1.4151(6)	0.2872(19)	10.00(18)	8*g*
	F_4	0.7042(7)	0.0232(8)	-1.2212(8)	0.2872(19)	10.00(18)	8*g*
	F_5	0.4964(4)	-0.1571(4)	-1.0288(5)	0.2872(19)	10.00(18)	8*g*
	Br_1	0.6605(6)	-0.0410(7)	-0.9845(6)	0.2872(19)	10.00(18)	8*g*
	C_1_2	0.4646(4)	-0.1095(5)	-0.3801(4)	0.2573(19)	10.0(2)	8*g*
	C_2_2	0.3898(7)	-0.0714(13)	-0.4287(4)	0.2573(19)	10.0(2)	8*g*
	C_3_2	0.3245(11)	-0.034(2)	-0.3617(6)	0.2573(19)	10.0(2)	8*g*
	C_4_2	0.3344(10)	-0.0336(18)	-0.2464(5)	0.2573(19)	10.0(2)	8*g*
	C_5_2	0.4094(6)	-0.0718(8)	-0.1984(4)	0.2573(19)	10.0(2)	8*g*
	C_6_2	0.4751(5)	-0.1102(7)	-0.2646(4)	0.2573(19)	10.0(2)	8*g*
	F_1_2	0.3804(9)	-0.0712(17)	-0.5394(5)	0.2573(19)	10.0(2)	8*g*

	F_2_2	0.2526(17)	0.003(3)	-0.4078(7)	0.2573(19)	10.0(2)	8*g*
	F_3_2	0.2717(15)	0.003(3)	-0.1823(7)	0.2573(19)	10.0(2)	8*g*
	F_4_2	0.4171(7)	-0.0710(9)	-0.0876(4)	0.2573(19)	10.0(2)	8*g*
	F_5_2	0.5261(6)	-0.1456(10)	-0.4463(4)	0.2573(19)	10.0(2)	8*g*
	Br_1_2	0.5765(11)	-0.163(2)	-0.1992(5)	0.2573(19)	10.0(2)	8*g*

Table S7. Summary of simulation parameters of EPR spectra for photocatalytic reaction with four different substrates catalysed by MFM-300(Cr).

**Adduct**	**g-factor**	**$A^{14}N$ / G**	**$A^{\beta}H$ / G**	**lw/mT**	**Weighting**
PBN*$^{\bullet}$Ph-OCH_3	2.00570	15.0	3.0	0.05<br>0.16	1
PBN*$^{\bullet}$Nap	2.00578	14.8	4.3	0.05<br>0.15	1
PBN*$^{\bullet}$Ph-$COCH_3$	2.00582	14.7	2.6	0.03<br>0.23	1
PBN*$^{\bullet}$$PhF_5$	2.00565	15.0	2.5	0.05<br>0.20	1

[*] lw is the homogeneous Lorentzian linewidth; *g* and *A* are the Hamiltonia g-tensor and hyperfine constant parameters, respectively.

Calculation of yields using ^{1}H NMR spectroscopy.

Yields of products were determined by ^{1}H NMR spectroscopy using nitromethane or cyclohexane as the internal standard, and yields were confirmed by integration of the signals. Determination of the yields by ^{1}H NMR spectroscopy was according to the equation below (take nitromethane as an example).

$$\text{Yield} = \frac{3}{x} * \left(\frac{\text{Area}_{\text{product}}}{\text{Area}_{\text{internal standard}}}\right)\left(\frac{n_{CH_3NO_2}}{n_{\text{theoretical product}}}\right) * 100\%$$

$\text{Area}_{\text{product}}$ and $\text{Area}_{\text{internal standard}}$ are the integration of the specific product peak and the internal standard peak, respectively. x is the proton number of the specific product peak. n_{CH3NO2} (mol) was calculated from the weight of added internal standard divided by the molecular weight. $n_{\text{theoretical product}}$ was calculated from the weight of added substrate divided by the corresponding molecular weight. The ^{1}H NMR and ^{13}C NMR spectra of reaction mixtures of **2a**, **2a'**, **4d** and **4d'** (both X/D and X/H exchanges) are given below.

^{1}H NMR spectrum of 2a' in Figure 3 for X/H exchange

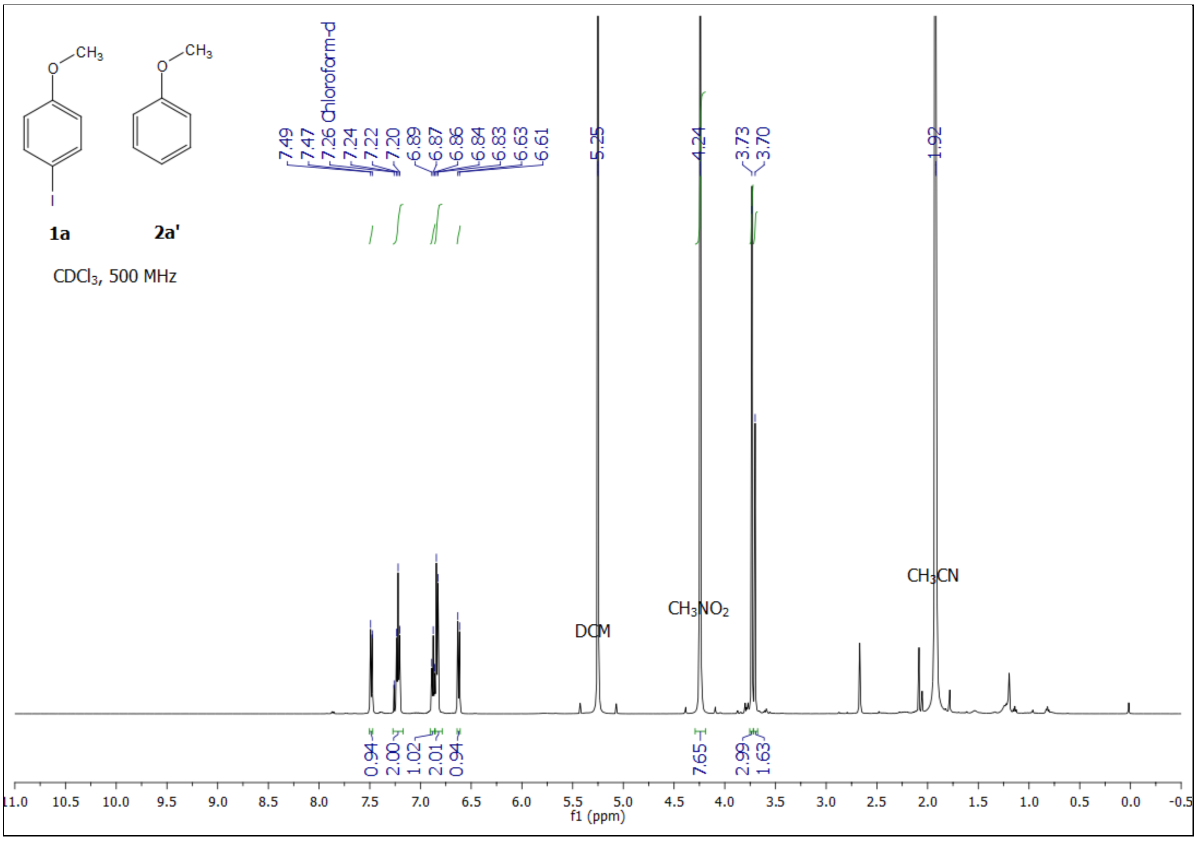

^{13}C NMR spectrum of 2a' in Figure 3 for X/H exchange.

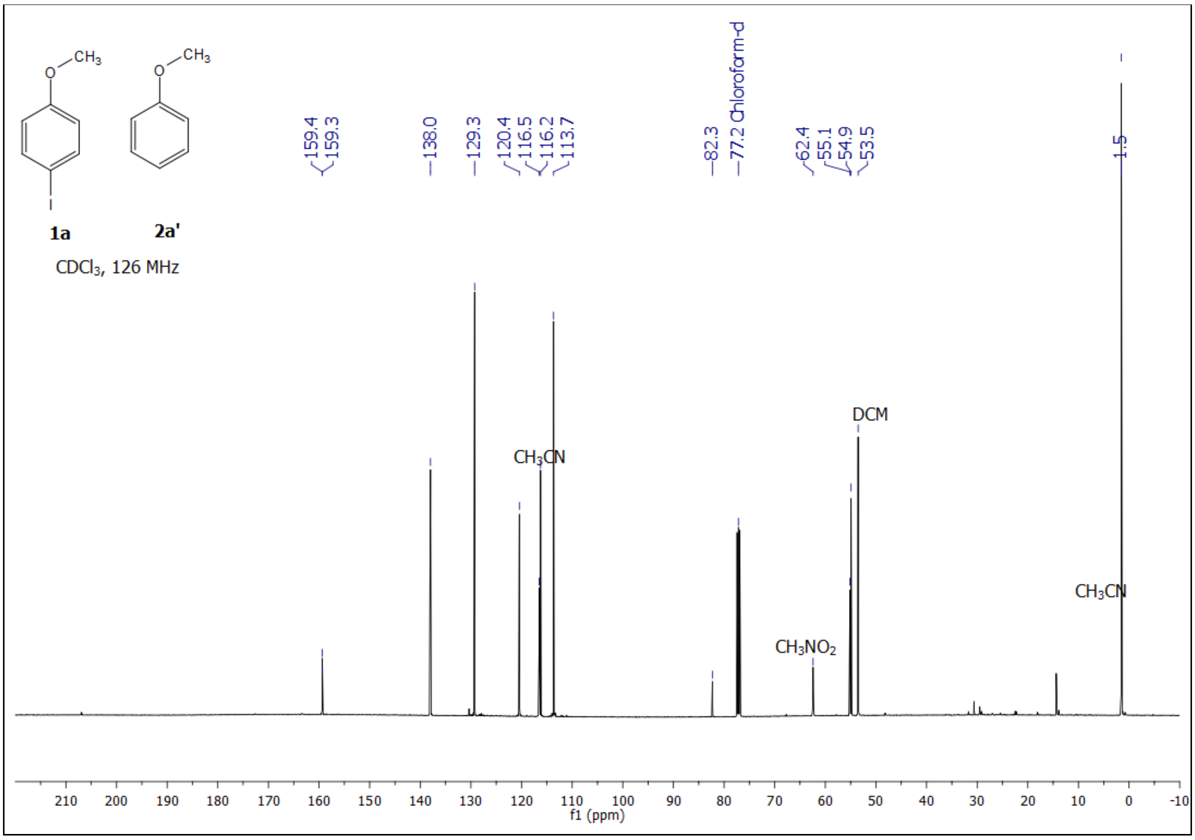

^{1}H NMR spectrum of 2a in Figure 3 for X/D exchange.

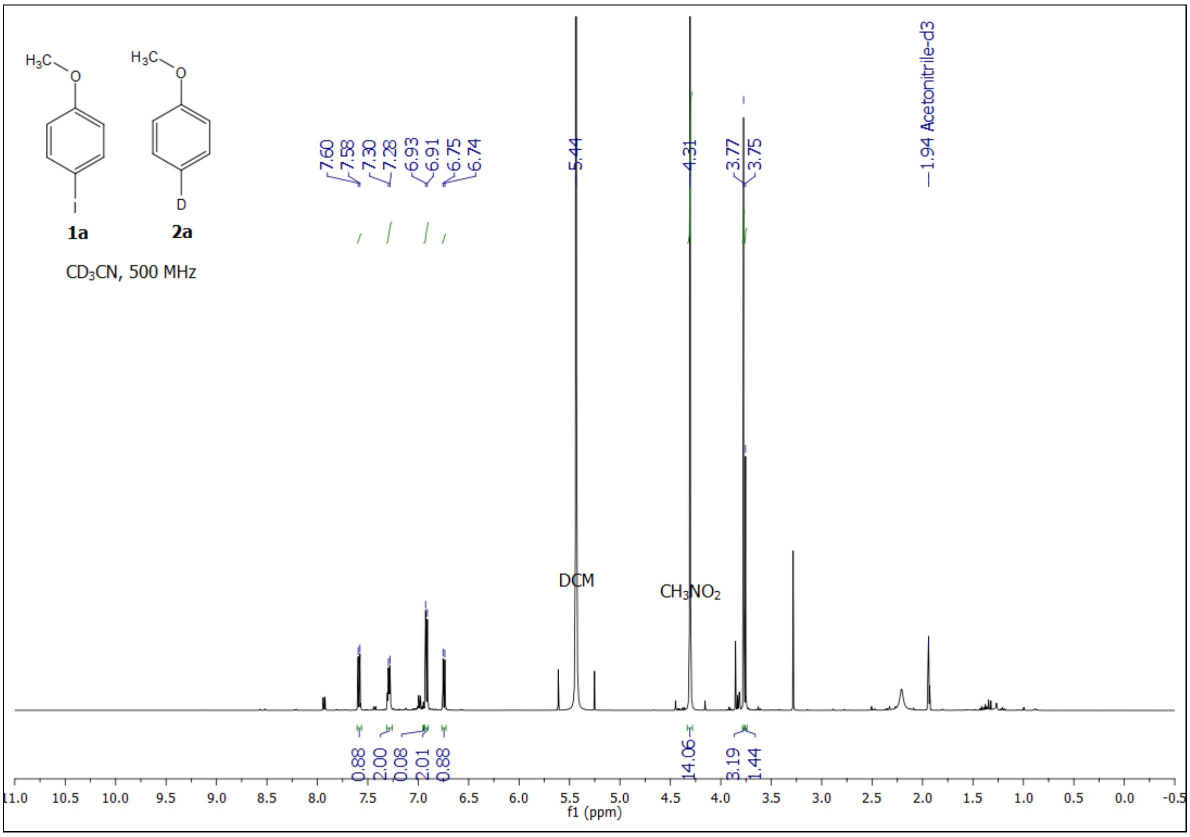

^{13}C NMR spectrum of 2a in Figure 3 for X/D exchange.

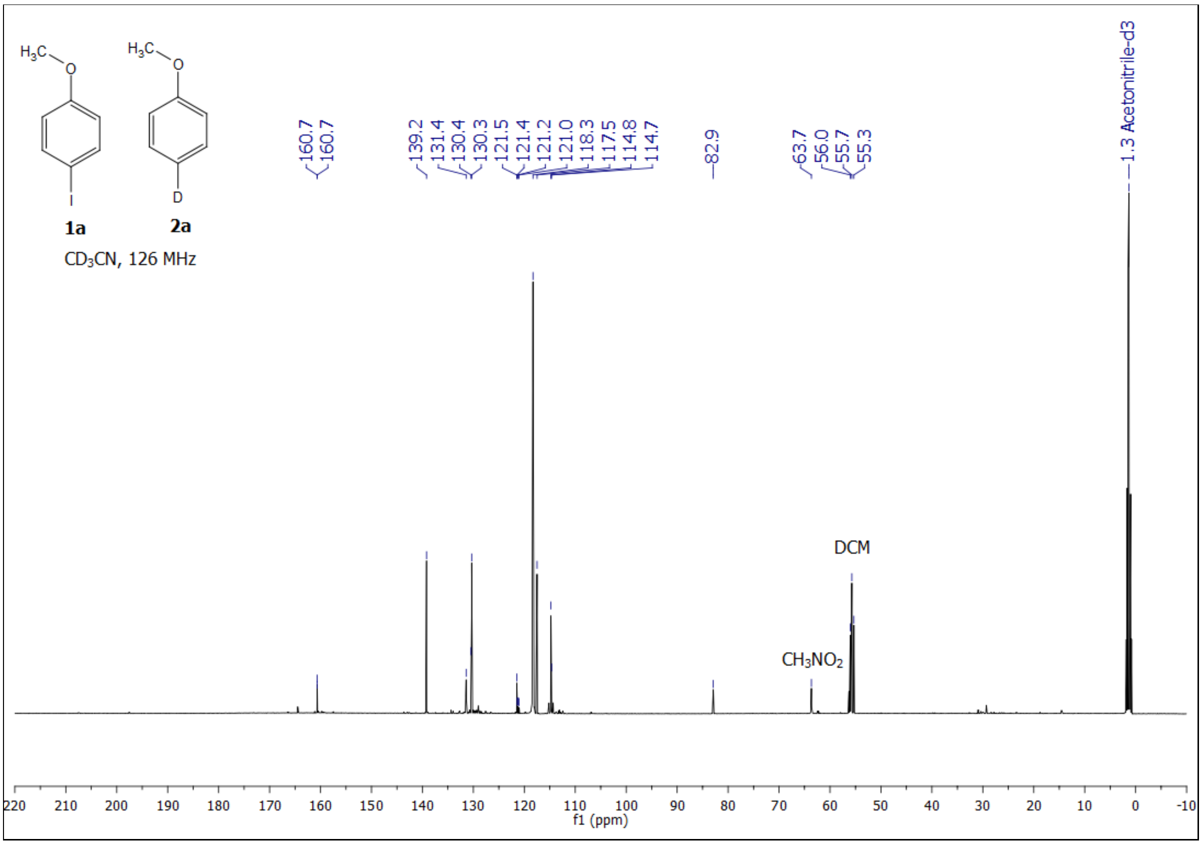

^{1}H NMR spectrum of 4d' in Figure 3 for X/H exchange.

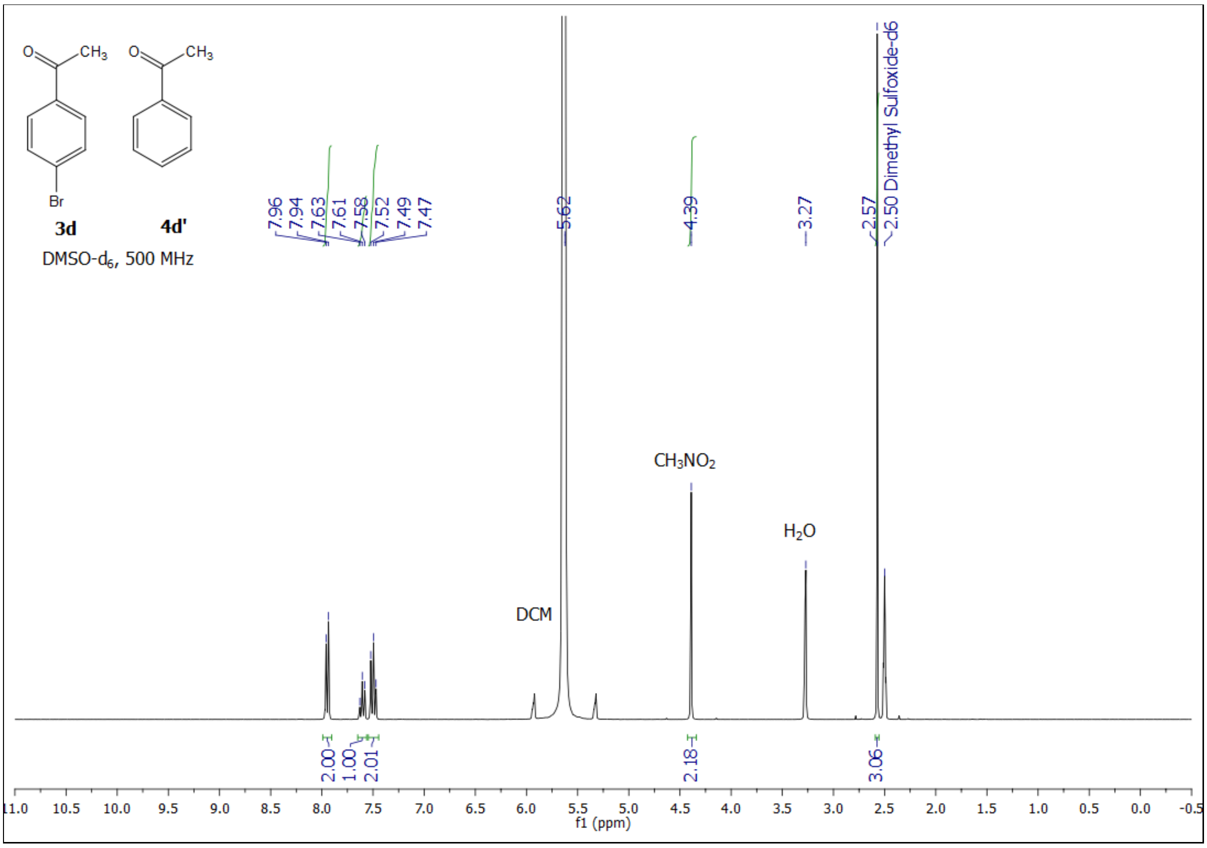

^{13}C NMR spectrum of 4d' in Figure 3 for X/H exchange.

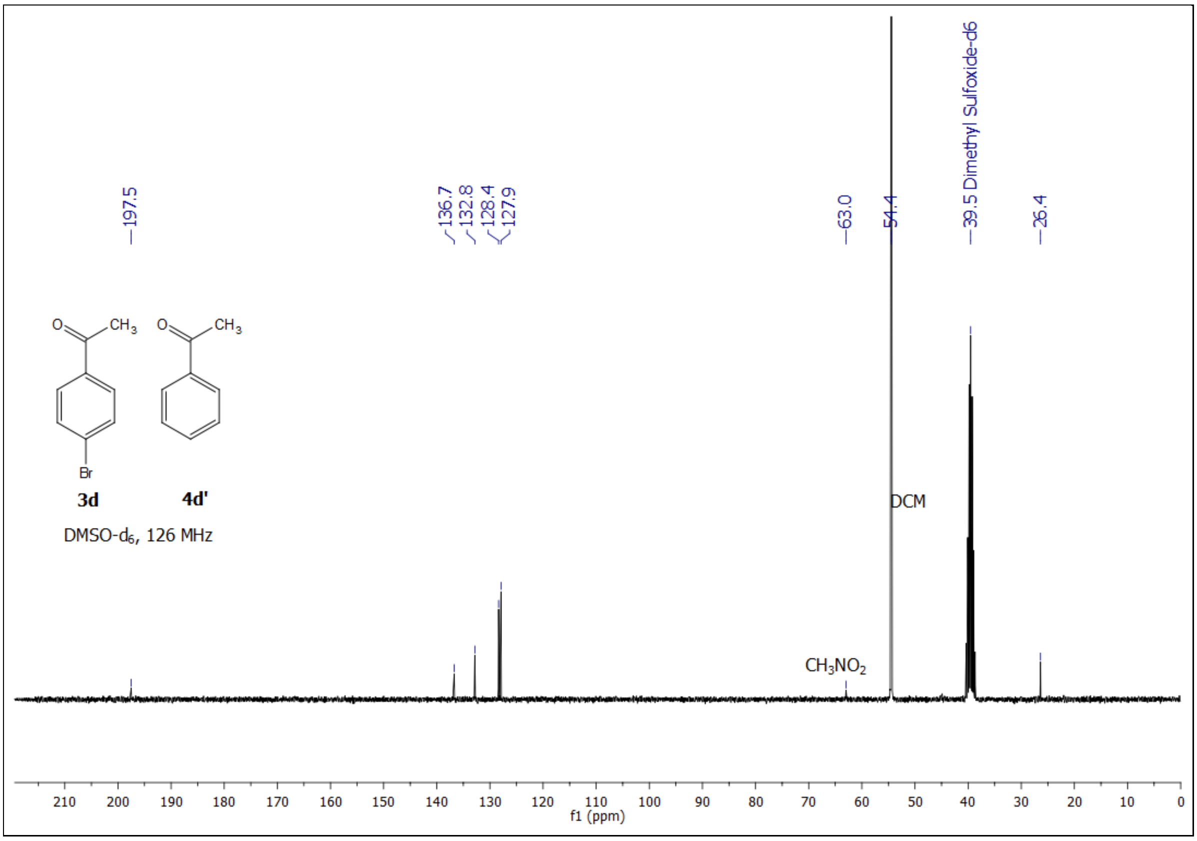

^{1}H NMR spectrum of 4d in Figure 3 for X/D exchange.

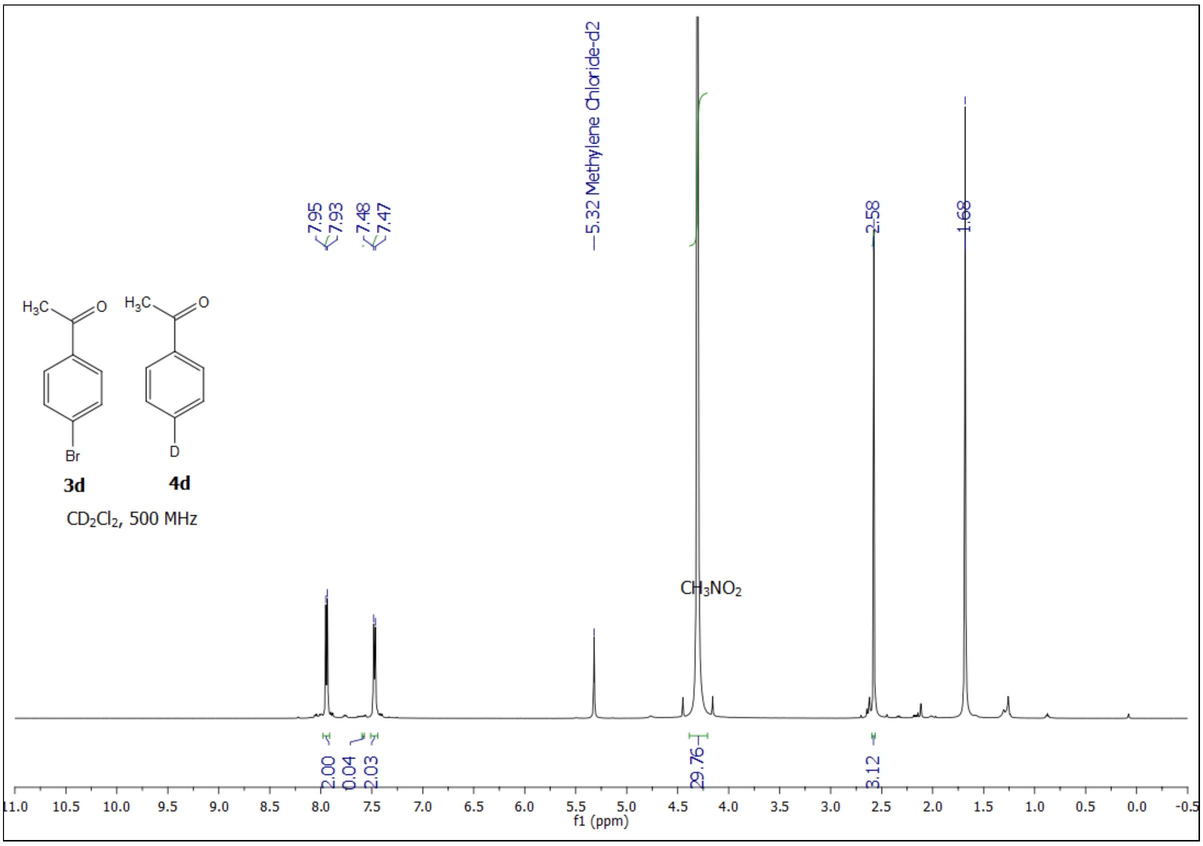

^{13}C NMR spectrum of 4d in Figure 3 for X/D exchange.

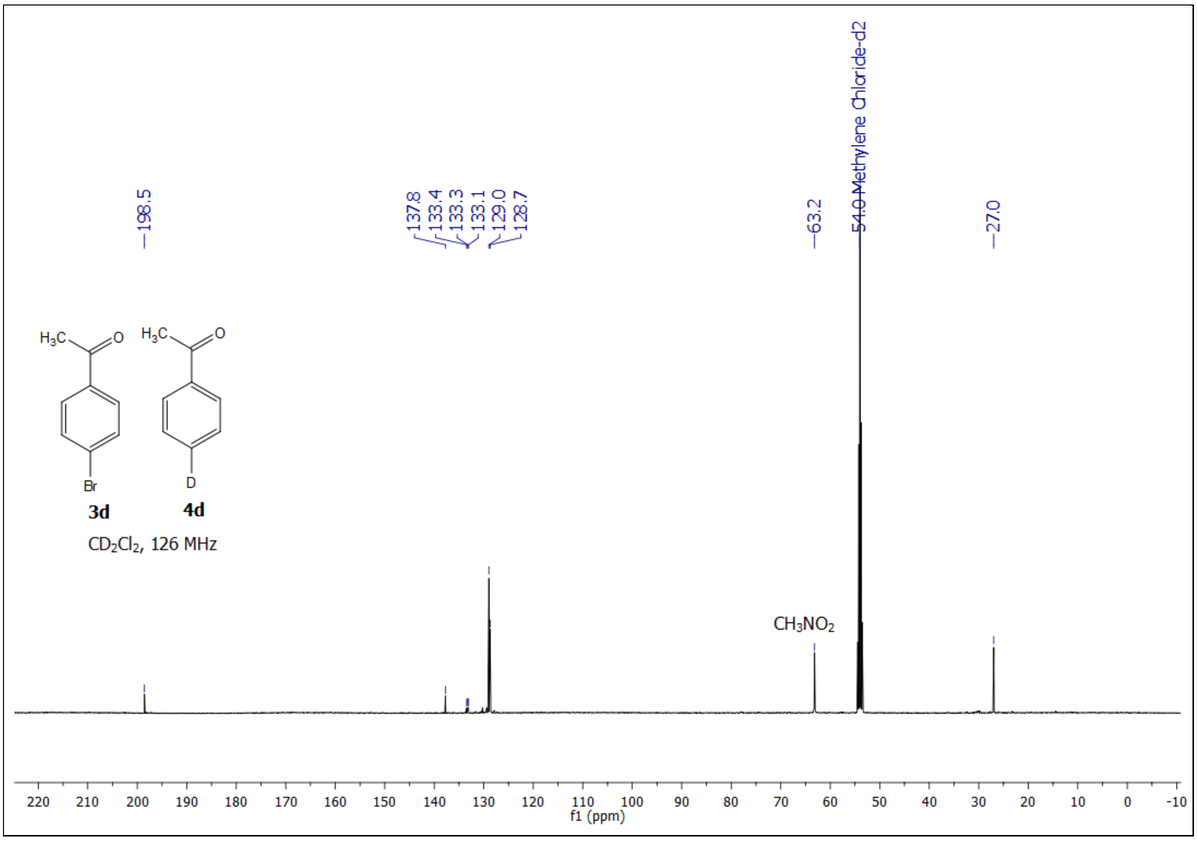

Spectroscopic Data for photocatalytic C-X/C-D and C-X/C-H exchange of halides.

(4-D)-anisol (**2a**, Figure 3) and anisole (**2a'**, Figure 3) [9,10]

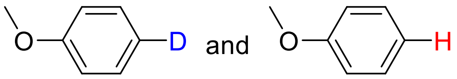

2a **2a'**

2a and **2a'** were prepared according to the general procedure using 4-iodoanisole (0.117 g, 0.500 mmol) as the starting material. **2a** and **2a'** were obtained in 66% (92% D) and 68% yield after 24 h.
^{1}H NMR of **2a** (500 MHz, CD_3CN) δ 7.27 (d, 2H), 6.90 (d, 2H), 3.72 (s, 3H);
^{13}C NMR of **2a** (126 MHz, CD_3CN) δ 160.5, 130.4, 121.2 (t, $J_{C\text{-}D}$ = 24.8 Hz), 114.8, 55.8.
^{1}H NMR of **2a'** (500 MHz, $CDCl_3$) δ 7.17 (m, 2H), 6.82 (t, 1H), 6.78 (d, 2H), 3.68 (s, 3H);
^{13}C NMR of **2a'** (126 MHz, $CDCl_3$) δ 159.2, 129.1, 120.3, 113.5, 54.7.

Benzene (X=H) and iodobenzene (X=I) (**2b'**, Figure 3) [11]

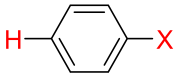

2b'

2b' was prepared according to the general procedure using 1, 4-diiodobenzene (0.165 g, 0.500 mmol) as the starting material, and obtained in 88% yield with 55% of iodobenzene (X=I) and 33% of benzene (X=H) after 48 h.

^{1}H NMR of benzene (500 MHz, $CDCl_3$) δ 7.30 (s, 6H);

^{13}C NMR of benzene (126 MHz, $CDCl_3$) δ 128.0.

^{1}H NMR of iodobenzene (500 MHz, $CDCl_3$) δ 7.61 (d, 2H); 7.26 (m, 1H), 7.03 (t, 2H).

^{13}C NMR of iodobenzene (126 MHz, $CDCl_3$) δ 137.0, 130.0, 127.2, 93.8

Aminobenzene (**2c'**, Figure 3) [12]

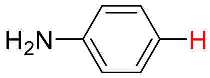

2c'

2c' was prepared according to the general procedure using 4-iodoaniline (0.110 g, 0.500 mmol) as the starting material, and obtained in 93% yield after 24 h.

^{1}H NMR of **2c'** (500 MHz, $CDCl_3$) δ 7.04 (m, 2H), 6.62 (m, 1H), 6.58 (m, 2H), 3.59 (br, 2H);

^{13}C NMR of **2c'** (126 MHz, $CDCl_3$) δ 146.6, 128.9, 117.7, 114.6.

(4-D)-benzonitrile (**2d**, Figure 3) and benzonitrile (**2d'**, Figure 3) [13]

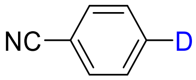

and
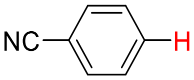

2d **2d'**

2d and **2d'** were prepared according to the general procedure using 4-iodobenzonitrile (0.115 g, 0.500 mmol) as the starting material. **2d** and **2d'** were obtained in >99% (92% D) and >99% yield after 24 h.

^{1}H NMR of **2d** (500 MHz, CD_3CN) δ 7.71 (m, 2H), 7.53 (m, 2H);

^{13}C NMR of **2d** (126 MHz, CD_3CN) δ 133.6 (t, $J_{C\text{-}D}$ = 24.3 Hz), 133.0, 130.0, 119.6, 112.9.

^{1}H NMR of **2d'** (500 MHz, $CDCl_3$) δ 7.61 (m, 2H), 7.57 (m, 1H), 7.43 (m, 2H);

^{13}C NMR of **2d'** (126 MHz, $CDCl_3$) δ 132.8, 132.0, 129.1, 118.8, 112.2.

(4-D)-benzaldehyde (**2e**, Figure 3) and benzaldehyde (**2e'**, Figure 3) [14,15]

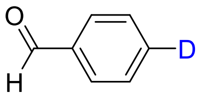

and
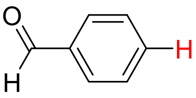

2e **2e'**

2e and **2e'** were prepared according to the general procedure using 4-iodobenzaldehyde (0.116 g, 0.500 mmol) as the starting material. **2e** and **2e'** were obtained in >99% (95% D) and >99% yield after 24 h.

^{1}H NMR of **2e** (500 MHz, CD_2Cl_2) δ 10.00 (s, 1H), 7.88 (m, 2H), 7.55 (m, 2H);

^{13}C NMR of **2e** (126 MHz, CD_2Cl_2) δ 192.8, 137.1, 134.6 (t, $J_{C\text{-}D}$ = 24.7 Hz), 130.1, 129.4.

^{1}H NMR of **2e'** (500 MHz, $CDCl_3$) δ 10.00 (br, 1H), 7.87 (d, J = 6.9 Hz, 2H), 7.63 (t, 1H), 7.52 (t, 2H);

^{13}C NMR of **2e'** (126 MHz, $CDCl_3$) δ 192.5, 136.5, 134.6, 129.8, 129.1.

(4-D)- acetophenone (**2f**, Figure 3) and acetophenone (**2f’**, Figure 3) [15–17]

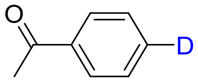

and

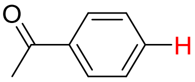

2f **2f'**

2f and **2f’** were prepared according to the general procedure using 4’-iodoacetophenone (0.123 g, 0.500 mmol) as the starting material. **2f** and **2f’** were obtained in >99% (97% D) and >99% yield after 24 h.
^{1}H NMR of **2f** (500 MHz, $CDCl_3$) δ 7.80 (m, 2H), 7.59 (m, 2H), 2.57 (s, 3H);
^{13}C NMR of **2f** (126 MHz, $CDCl_3$) δ 197.1, 135.9, 132.0, 129.9, 128.4, 26.6.
^{1}H NMR of **2f’** (500 MHz, $CDCl_3$) δ 7.93 (d, 2H), 7.53 (t, 1H), 7.42 (d, 2H), 2.57 (s, 3H);
^{13}C NMR of **2f’** (126 MHz, $CDCl_3$) δ 198.1, 137.1, 133.1, 128.6, 128.3, 26.6.
Spectral data are in accordance with previous reports.

Benzoic acid (**2g’**, **2h’**, **2i’**, Figure 3) [13,18]

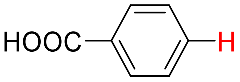

2g'

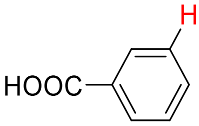

2h'

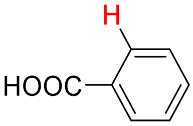

2i'

2g’, **2h’** and **2i’** were prepared using 4-iodobenzoic acid (0.124 g, 0.500 mmol), 3-iodobenzoic acid (0.1240 g, 0.5 mmol) and 2-iodobenzoic acid (0.1240 g, 0.5 mmol) as the starting material, respectively. After the catalyst MOF was separated by centrifugation, HCl (6M) was added dropwise into the mixture solution till the pH value to 5-7. The other post-processing is the same as the general procedure. **2g’**, **2h’** and **2i’** were obtained in 89%, 70% and 83% yield after 24 h separately.
^{1}H NMR of **2g’**, **2h’** or **2i’** (500 MHz, $CDCl_3$) δ 7.93 (m, 2H), 7.48 (m 1H), 7.35 (m, 2H);
^{13}C NMR of **2g’**, **2h’** or **2i’** (126 MHz, $CDCl_3$) δ 168.7, 133.0, 131.0, 129.5, 128.1.

(4-D)-methyl benzoate (**2j**, Figure 3) and methyl benzoate (**2j’**, Figure 3) [13,16,19]

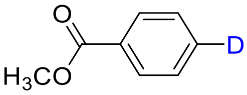

and

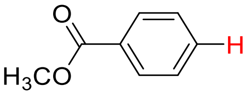

2j **2j'**

2j and **2j’** were prepared according to the general procedure using methyl 4-iodobenzoate (0.131 g, 0.500 mmol) as the starting material. **2j** and **2j’** were obtained in 56% (90% D) and 62% yield after 24 h.
^{1}H NMR of **2j** (500 MHz, CD_3CN) δ 7.97 (m, 2H), 7.48 (m, 2H), 3.84 (s, 3H);
^{13}C NMR of **2j** (126 MHz, CD_3CN) δ 168.4, 131.9, 130.6 (t, $J_{C\text{-}D}$ = 40.2 Hz), 130.2, 129.5, 53.0.
^{1}H NMR of **2j’** (500 MHz, $CDCl_3$) δ 7.88 (m, 2H), 7.45 (m, 1H), 7.32 (m, 2H), 3.77 (s, 3H);
^{13}C NMR of **2j’** (126 MHz, $CDCl_3$) δ 166.5, 132.6, 130.6, 129.0, 128.0, 51.6.

(4-D)-1-methoxycarbonyl-3-methylbenzene (**2k**, Figure 3) and 1-methoxycarbonyl-3-methylbenzene (**2k'**, Figure 3) [20]

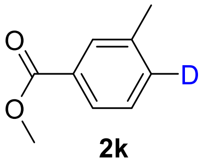

and

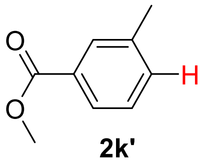

2k **2k'**

2k and **2k'** were prepared according to the general procedure using methyl 4-iodo-3-methylbenzoate (0.138 g, 0.500 mmol) as the starting material. **2k** and **2k'** were obtained in 64% (26% D) and 69% yield after 24 h.

^{1}H NMR of **2k** (500 MHz, CD_3CN) δ 7.79 (s, 1H), 7.76 (m, 1H), 7.35 (m, 1H), 3.83 (s, 3H), 2.35 (s, 3H);

^{13}C NMR of **2k** (126 MHz, CD_3CN) δ 168.5, 138.6, 134.6 (t, $J_{C\text{-}D}$ = 24.3 Hz), 130.6, 129.5, 128.8, 127.4, 52.9, 27.5.

^{1}H NMR of **2k'** (500 MHz, $CDCl_3$) δ 7.62 (m, 1H), 7.60 (m, 1H), 7.20 (m, 1H), 7.14 (m, 1H), 3.68 (s, 3H), 2.20 (s, 3H);

^{13}C NMR of **2k'** (126 MHz, $CDCl_3$) δ 166.5, 138.7, 133.2, 129.7, 129.3, 127.8, 126.0, 51.4, 20.4.

Ethyl benzoate (**2l'**, Figure 3) [19,21]

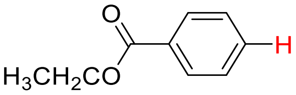

2l'

2l' was prepared according to the general procedure using ethyl 4-iodobenzoate (0.138 g, 0.500 mmol) as the starting material, and obtained in 79% yield after 24 h.

^{1}H NMR of **2l'** (500 MHz, $CDCl_3$) δ 7.94 (m, 2H), 7.48 (m, 1H), 7.37 (m, 2H), 4.26 (q, J = 6.9 Hz, 2H), 1.30 (t, J = 7.1 Hz, 3H);

^{13}C NMR of **2l'** (126 MHz, $CDCl_3$) δ 165.9, 132.4, 130.4, 128.8, 127.9, 60.4, 13.6.

(4-D)-acetanilide (**2m**, Figure 3) and acetanilide (**2m'**, Figure 3) [22]

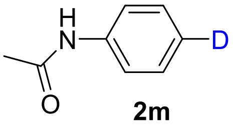

and

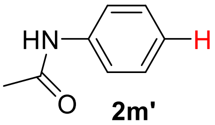

2m **2m'**

2m and **2m'** were prepared according to the general procedure using 4'-iodoacetanilide (0.131 g, 0.500 mmol) as the starting material. **2m** and **2m'** were obtained in 56% (94% D) and 67% yield after 24 h.

^{1}H NMR of **2m** (500 MHz, CD_3CN) δ 7.48 (m, 2H), 7.29 (m, 2H), 2.05 (s, 3H);

^{13}C NMR of **2m** (126 MHz, CD_3CN) δ 170.7, 138.7, 129.0, 124.1 (t, $J_{C\text{-}D}$ = 24.2 Hz), 120.2, 23.4.

^{1}H NMR of **2m'** (500 MHz, $CDCl_3$) δ 8.31 (br, 1H), 7.39 (m, 2H), 7.14 (m, 2H), 6.92 (m, 1H), 1.97 (s, 3H);

^{13}C NMR of **2m'** (126 MHz, $CDCl_3$) δ 168.7, 137.2, 128.3, 123.4, 119.4, 23.6.

(4-D)-N-Boc-aniline (**2n**, Figure 3) and N-Boc-aniline (**2n'**, Figure 3) [23,24]

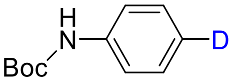

and

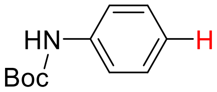

2n **2n'**

2n and **2n'** were prepared according to the general procedure using N-*tert*-butoxycarbonyl)-4-iodoaniline (0.160 g, 0.500 mmol) as the starting material. **2n** and **2n'** were obtained in 70% (94% D)

and 72% yield after 24 h. After purification by preparative-scale high performance liquid chromatography (HPLC), **2n’** was isolated 66.7 mg (69% yield) as a white solid. The ^{1}H and ^{13}C NMR spectra of **2n’** are given in next section.
^{1}H NMR of **2n** (500 MHz, CD_3CN) δ 7.34 (m, 2H), 7.25 (m, 2H), 1.44 (s, 9H);
^{13}C NMR of **2n** (126 MHz, $CDCl_3$) δ 152.9, 138.5, 129.0, 122.9 (t, $J_{C\text{-}D}$ = 30.9 Hz), 118.7, 80.6, 28.5.
^{1}H NMR of **2n’** (500 MHz, $CDCl_3$) δ 7.28 (m, 2H), 7.21 (m, 2H), 6.96 (m, 1H), 1.45 (s, 9H);
^{13}C NMR of **2n’** (126 MHz, $CDCl_3$) δ 152.9, 138.5, 129.1, 123.2, 118.7, 80.6, 28.5.

(4-D)-2-fluoroaniline (**2o**, Figure 3) and 2-fluoroaniline (**2o’**, Figure 3) [22]

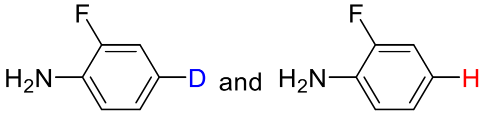

2o **2o'**

2o and **2o’** were prepared according to the general procedure using 2-fluoro-4-iodoaniline (0.119 g, 0.500 mmol) as the starting material. **2o** and **2o’** were obtained in 70% (63% D) and 74% yield after 24 h.
^{1}H NMR of **2o** (500 MHz, CD_3CN) δ 6.96-6.86 (m, 2H), 6.79 (m, 1H), 6.62 (m, 0.37H);
^{13}C NMR of **2o** (126 MHz, CD_3CN) δ 152.5 (d, $J_{C\text{-}F}$ = 234.1 Hz), 136.3 (d, $J_{C\text{-}F}$ = 12.8 Hz), 125.5 (d, $J_{C\text{-}F}$ = 3.4 Hz), 119.6 (d, J = 4.3 Hz), 118.1 (d, J = 3.9 Hz), 115.7 (d, J = 18.6 Hz).
^{19}F NMR of **2o** (471 MHz, CD_3CN) δ -136.7.
^{1}H NMR of **2o’** (500 MHz, $CDCl_3$) δ 6.83-6.72 (m, 2H), 6.64 (m, 1H), 6.49 (m, 1H), 3.76 (br, 2H);
^{13}C NMR of **2o’** (126 MHz, $CDCl_3$) δ 151.1 (d, $J_{C\text{-}F}$ = 237.7 Hz), 134.6 (d, $J_{C\text{-}F}$ = 12.9 Hz), 124.1 (d, $J_{C\text{-}F}$ = 3.5 Hz), 118.1 (d, $J_{C\text{-}F}$ = 4.1 Hz), 117.7 (d, J = 6.8 Hz), 114.6 (d, J = 18.4 Hz).
^{19}F NMR of **2o’** (471 MHz, $CDCl_3$) δ -136.2.

3-Phenylpropanoic acid methyl ester (**2p’**, Figure 3) [25]

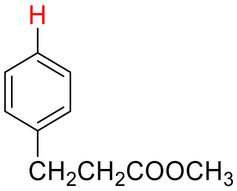

2p'

2p’ was prepared according to the general procedure using methyl 3-(4-iodophenyl) propanoate (0.145 g, 0.500 mmol) as the starting material, and obtained in 91% yield after 24 h.
^{1}H NMR of **2p’** (500 MHz, $CDCl_3$) δ 7.14 (m, 2H), 7.05 (m, 3H), 3.51 (s, 3H), 2.79 (t, J = 7.8 Hz, 2H), 2.48 (t, J = 7.8 Hz, 2H);
^{13}C NMR of **2p’** (126 MHz, $CDCl_3$) δ 172.9, 140.2, 128.1, 127.9, 125.9, 51.1, 35.2, 30.4.

(1-D)-naphthalene (**2q**, Figure 3) and naphthalene (**2q’**, Figure 3) [13]

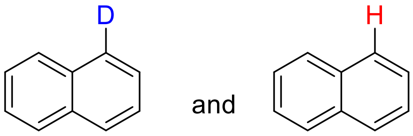

2q **2q'**

2q and **2q’** were prepared according to the general procedure using 1-iodonaphthalene (0.127 g, 0.500 mmol) as the starting material. **2q** and **2q’** were obtained in 90% (96% D) and >99% yield after 24 h.

After purification by preparative-scale high performance liquid chromatography (HPLC), **2q** was isolated 56.8 mg (88% yield) as a white solid. The ^{1}H and ^{13}C NMR spectra are given in next section.
^{1}H NMR of **2q** (500 MHz, $CDCl_3$) δ 7.86 (m, 3H), 7.50 (m, 4H);
^{13}C NMR of **2q** (126 MHz, $CDCl_3$) δ 133.6, 128.0, 126.0.
^{1}H NMR of **2q’** (500 MHz, $CDCl_3$) δ 7.89 (m, 4H), 7.52 (m, 4H);
^{13}C NMR of **2q’** (126 MHz, $CDCl_3$) δ 133.6, 128.0, 126.0.

(2-D)-naphthalene (**2r**, Figure 3) and naphthalene (**2r’**, Figure 3) [13]

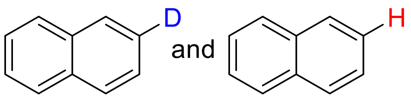

2r **2r'**

2r and **2r’** were prepared according to the general procedure using 2-iodonaphthalene (0.127 g, 0.500 mmol) as the starting material. **2r** and **2r’** were obtained in 65% (95% D) and 73% yield after 24 h. After purification by preparative-scale high performance liquid chromatography (HPLC), **2r’** was isolated 44.9 mg (70% yield) as a white solid. The ^{1}H and ^{13}C NMR spectra are given in next section.
^{1}H NMR of **2r** (500 MHz, $CDCl_3$) δ 7.90 (m, 4H), 7.51 (m, 3H);
^{13}C NMR of **2r** (126 MHz, $CDCl_3$) δ 133.6, 128.0, 127.9, 125.9, 125.8, 125.7 (t, J = 24.8 Hz).
^{1}H NMR and ^{13}C NMR data of **2r’** were the same as previously described.

(6-D)-methyl 3-bromobenzoate (**2d**, Figure 3) and methyl 3-bromobenzoate (**2d’**, Figure 3) [19]

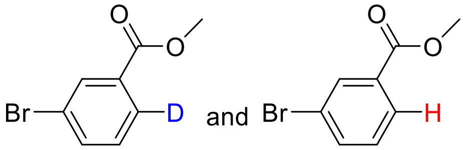

2s **2s'**

2s and **2s’** were prepared according to the general procedure using methyl 5-bromo-2-iodobenzoate (0.171 g, 0.500 mmol) as the starting material. **2s** and **2s’** were obtained in 78% (96% D) and 83% yield after 24 h.
^{1}H NMR of **2s** (500 MHz, CD_3CN) δ 8.10 (m, 1H), 7.76 (m, 1H), 7.40 (m, 1H), 3.85 (s, 3H);
^{13}C NMR of **2s** (126 MHz, CD_3CN) δ 167.0, 137.0, 133.1, 132.9, 131.5, 128.9 (t, $J_{C\text{-}D}$ = 25.3 Hz), 122.9, 53.3.
^{1}H NMR of **2s’** (500 MHz, $CDCl_3$) δ 7.91 (m, 1H), 7.74 (m, 1H), 7.50 (m, 1H), 7.16 (m, 1H), 3.68 (s, 3H);
^{13}C NMR of **2s’** (126 MHz, $CDCl_3$) δ 165.0, 135.3, 131.6, 131.6, 129.7, 127.5, 121.6, 51.7.

(1-D)-2,3,4,5,6-pentafluorobenzene (**2t**, Figure 3) and pentafluorobenzene (**2t’**, Figure 3) [26][27]

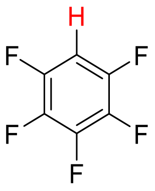

2t'

2t’ was prepared according to the general procedure using iodopentafluorobenzene (0.147 g, 0.500 mmol) as the starting material and obtained in >99% yield after 24 h.
^{1}H NMR of **2t’** (500 MHz, $CDCl_3$) δ 6.87 (m, 1H);

^{13}C NMR of **2t’** (126 MHz, $CDCl_3$) δ 146.2 (m), 141.5 (m), 137.5 (m), 102.3 (t, *J* = 23.6 Hz);
^{19}F NMR of **2t’** (471 MHz, $CDCl_3$) δ -140.3 (m), -155.8 (t, *J* = 19.9 Hz), -163.9 (m).

(3,6-D2)-1,2,4,5-tetrafluorobenzene (**2u**, Figure 3) and 1,2,4,5-tetrafluorobenzene (**2u’**, Figure 3) [28,29]

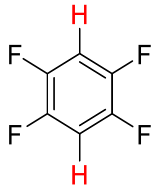

2u'

2u’ was prepared according to the general procedure using 1, 4-diiodotetrafluorobenzene (0.201 g, 0.500 mmol) as the starting material and obtained in >99% yield after 24 h.
^{1}H NMR of **2u’** (500 MHz, $CDCl_3$) δ 7.01 (m, 2H);
^{13}C NMR of **2u’** (126 MHz, $CDCl_3$) δ 145.4(m), 106.3 (m);
^{19}F NMR of **2u’** (471 MHz, $CDCl_3$) δ -139.8 (t, *J* = 8.7 Hz).

2-Chloropyridine (**2v’**, Figure 3)[30]

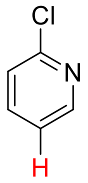

2v'

2v’ was prepared according to the general procedure using 2-choro-5-iodopyridine (0.120 g, 0.500 mmol) as the starting material and obtained in 36% yield after 24 h.
^{1}H NMR of **2v’** (500 MHz, DMSO-d_6) δ 8.41 (m, 1H), 7.85 (m, 1H), 7.49 (m, 1H), 7.39 (m, 1H);
^{13}C NMR of **2v’** (126 MHz, $CDCl_3$) δ. 150.6, 149.5, 138.8, 124.3, 122.2.

Ethylbenzene (**2w’**, Figure 3) [31]

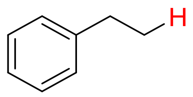

2w'

2w’ was prepared according to the general procedure using (2-iodoethyl) benzene (0.116 g, 0.500 mmol) as the starting material, and obtained in 84% yield after 48 h.
^{1}H NMR of **2w’** (500 MHz, $CDCl_3$) δ 7.07 (m, 5H), 2.50 (q, *J* = 7.7 Hz, 2H), 1.08 (t, *J* = 7.6 Hz, 3H);
^{13}C NMR of **2w’** (126 MHz, $CDCl_3$) δ 140.2, 127.9, 127.5, 125.2, 28.4, 15.2.

(4-D)-pyridine (**2x**, Figure 3) and pyridine (**2x’**, Figure 3) [28]

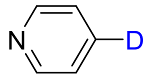 and 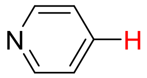

2x **2x'**

2x and **2x’** were prepared according to the general procedure using 4-iodopyridine (0.103 g, 0.500 mmol) as the starting material. **2x** and **2x’** were obtained in 35% (92% D) and 32% yield after 24 h.
^{1}H NMR of **2x** (500 MHz, CD_3CN) δ 8.50 (m, *J* = 5.7 Hz, 2H), 7.37 (m, *J* = 4.8 Hz, 2H);

^{13}C NMR of **2x** (126 MHz, CD_3CN) δ 150.0, 140.8 (t, $J_{C\text{-}D}$ = 25.2 Hz), 125.1.
^{1}H NMR of **2x'** (500 MHz, $CDCl_3$) δ 8.42 (m, 2H), 7.55 (m, 1H), 7.15 (m, 2H);
^{13}C NMR of **2x'** (126 MHz, $CDCl_3$) δ 149.2, 135.6, 123.4.

(2-D)-thiophen (**2y**, Figure 3) and thiophen (**2y'**, Figure 3) [32][33]

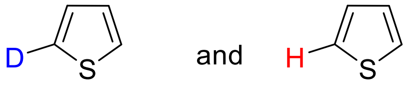

2y **2y'**

2y and **2y'** were prepared according to the general procedure using 2-iodothiophene (0.105 g, 0.500 mmol) as the starting material. **2y** and **2y'** were obtained in 33% (68% D) and 24% yield after 48 h.
^{1}H NMR of **2y** (500 MHz, CD_3CN) δ 7.48 (m, 2H), 7.41 (m, 1H);
^{13}C NMR of **2y** (126 MHz, CD_3CN) δ 128.0, 126.3, 126.2 (t, $J_{C\text{-}D}$ = 15.4 Hz).
^{1}H NMR of **2y** (500 MHz, CD_3CN) δ 7.41 (m, 1H), 7.12 (m, 2H);
^{13}C NMR of **2y** (126 MHz, CD_3CN) δ 128.0, 126.3, 126.2 (t, $J_{C\text{-}D}$ = 15.4 Hz).

(4-D)-benzaldehyde (**4a**, Figure 3) and benzaldehyde (**4a'**, Figure 3) [14,15]

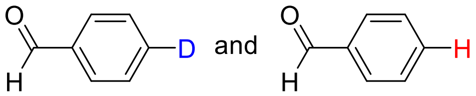

4a **4a'**

4a and **4a'** were prepared according to the general procedure using 4-bromobenzaldehyde (0.0925 g, 0.500 mmol) as the starting material. **4a** and **4a'** were obtained in 94% (86% D) and 95% yield after 24 h.
^{1}H NMR and ^{13}C NMR data were the same as previously described.

Benzaldehyde (**4d'**, Figure 3) [15]

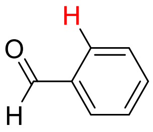

4b'

4b' was prepared according to the general procedure using 2-bromobenzaldehyde (0.0925 g, 0.500 mmol) as the starting materials, and obtained in 77% yield after 24 h.
^{1}H NMR and ^{13}C NMR data were the same as previously described.

Benzaldehyde (**4c'**, Figure 3) [15]

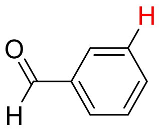

4c'

4c' was prepared according to the general procedure using 3-bromobenzaldehyde (0.0925 g, 0.500 mmol) as the starting materials, and obtained in 81% yield after 24 h.
^{1}H NMR and ^{13}C NMR data were the same as previously described.

(4-D)- acetophenone (**4d**, Figure 3) and acetophenone (**4d'**, Figure 3) [15–17]

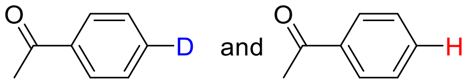

4d **4d'**

4d and **4d’** were prepared according to the general procedure using 4’-bromoacetophenone (0.0995 g, 0.500 mmol) as the starting material. **4d** and **4d’** were obtained in >99% (96% D) and >99% yield after 24 h.

^{1}H NMR and ^{13}C NMR data were the same as previously described.

After purification by preparative-scale high performance liquid chromatography (HPLC), **4d** was isolated 57.5 mg (95% yield) as a light-yellow oil. The ^{1}H and ^{13}C NMR spectra are given in next section.

Acetophenone (**4e’**, Figure 3) [15]

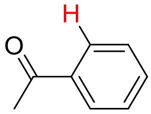

4e'

4e’ was prepared according to the general procedure using 2’-bromoacetophenone (0.0995 g, 0.500 mmol) as the starting materials, and obtained in 91% yield after 24 h.

^{1}H NMR and ^{13}C NMR data were the same as previously described.

Acetophenone (**4f’**, Figure 3) [15]

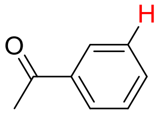

4f'

4f’ was prepared according to the general procedure using 3’-bromoacetophenone (0.0995 g, 0.500 mmol) as the starting materials, and obtained in 99%yield after 24 h.

^{1}H NMR and ^{13}C NMR data were the same as previously described.

After purification by preparative-scale high performance liquid chromatography (HPLC), **4f’** was isolated 58.3 mg (97% yield) as a light-yellow oil. The ^{1}H and ^{13}C NMR spectra are given in next section.

4-Acetylbiphenyl (**4g’**, Figure 3) [34]

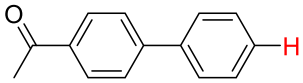

4g'

4g’ was prepared according to the general procedure using 4’-(4-bromophenyl) acetophenone (0.138 g, 0.500 mmol) as the starting material, and obtained in 51% yield after 24 h.

^{1}H NMR of **4g’** (500 MHz, DMSO-*d6*) δ 8.04 (m, 2H), 7.80 (m, 2H), 7.73 (m, 2H), 7.51 (m, 3H), 2.61 (s, 3H);

^{13}C NMR of **4g’** (126 MHz, DMSO-*d6*) δ 197.3, 144.8, 139.0, 135.2, 128.4, 128.3, 127.7, 126.5, 126.5, 25.8.

Benzonitrile (**4h’**, Figure 3)[13]

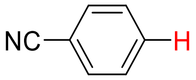

4h'

4h’ was prepared according to the general procedure using 4-bromobenzonitrile (0.0910 g, 0.500 mmol) as the starting material, and obtained in 55% yield after 24 h.
^{1}H NMR and ^{13}C NMR data were the same as previously described.

3-Fluorobenzonitrile (**4i’**, Figure 3) [35][36]

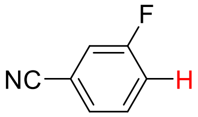

4i'

4i’ was prepared according to the general procedure using 3-bromo-5-fluorobenzonitrile (0.100 g, 0.500 mmol) as the starting materials, and obtained in 95% yield after 24 h.
^{1}H NMR of **4i’** (500 MHz, $CDCl_3$) δ 7.36 (m, 2H), 7.22 (m, 2H);
^{13}C NMR of **4i’** (126 MHz, $CDCl_3$) δ 161.7 (d, J_{C-F} = 249.2 Hz), 131.1 (d, J_{C-F} = 8.4 Hz), 128.0 (d, J_{C-F} = 3.5 Hz), 120.2 (d, J_{C-F} = 21.0 Hz), 118.7 (d, J_{C-F} = 24.8 Hz), 117.1 (d, J_{C-F} = 3.1 Hz), 113.2 (d, J = 9.5 Hz).
^{19}F NMR of **4i’** (471 MHz, $CDCl_3$) δ -110.54.

3-Fluorobenzonitrile (**4j’**, Figure 3) [35][36]

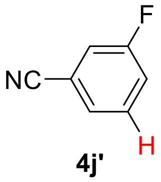

4j'

4j’ was prepared according to the general procedure using 4-bromo-3-fluorobenzonitrile (0.100 g, 0.500 mmol) as the starting materials, and obtained in >99% yield after 24 h.
^{1}H NMR and ^{13}C NMR data were the same as previously described.

Pentafluorobenzene (**4k’**, Figure 3) [26][27]

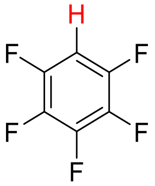

4k'

4k’ was prepared according to the general procedure using bromopentafluorobenzene (0.124 g, 0.500 mmol) as the starting material, and obtained in 99% yield after 24 h.
^{1}H NMR and ^{13}C NMR data were the same as previously described.

1,2,4,5-tetrafluorobenzene (**4l’**, Figure 3) [28,29]

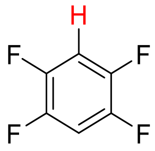

4l'

4l’ was prepared according to the general procedure using 3-bromo-1,2,4,5-tetrafluorobenzene (0.115 g, 0.500 mmol) as the starting materials, and obtained in 83% yield after 24 h.
^{1}H NMR and ^{13}C NMR data were the same as previously described.

1,2,4,5-tetrafluorobenzene (**4m’**, Figure 3) [28,29]

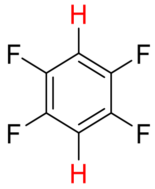

4m'

4m’ was prepared according to the general procedure using 1,4-dibromotetrafluorobenzene (0.154 g, 0.500 mmol) as the starting materials, and obtained in 76% yield after 24 h.
^{1}H NMR and ^{13}C NMR data were the same as previously described.

2,2',3,3',5,5',6,6'-Octafluoro-1,1'-biphenyl (**4n’**, Figure 3) [37][38]

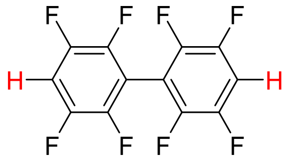

4n'

4n’ was prepared according to the general procedure using 4, 4’-dibromooctafluorobiphenyl (0.228 g, 0.500 mmol) as the starting material, and obtained in 92% yield after 24 h.
After purification by preparative-scale high performance liquid chromatography (HPLC), **4n’** was isolated 134 mg (90% yield) as a white solid. The ^{1}H and ^{13}C NMR spectra are given in next section.
^{1}H NMR of **4n’** (500 MHz, $CDCl_3$) δ 7.23 (m, 2H);
^{13}C NMR of **4n’** (126 MHz, $CDCl_3$) δ 146.7 (m), 144.7 (m), 142.6 (m), 107.8 (t, J = 22.6 Hz);
^{19}F NMR of **4n’** (471 MHz, $CDCl_3$) δ -138.2 (m), -138.8 (m).

Acetophenone (**4o’**, Figure 3) [15,39,40]

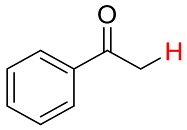

4o'

4o’ was prepared according to the general procedure using α-bromoacetophenone (0.0995 g, 0.500 mmol) as the starting material, and obtained in 44% yield with 46% by-product of 1, 4-diphenylbutane-1, 4-dione after 24 h.
^{1}H NMR and ^{13}C NMR of **4o’** were the same with **4e’**.

NMR spectra of isolated products.

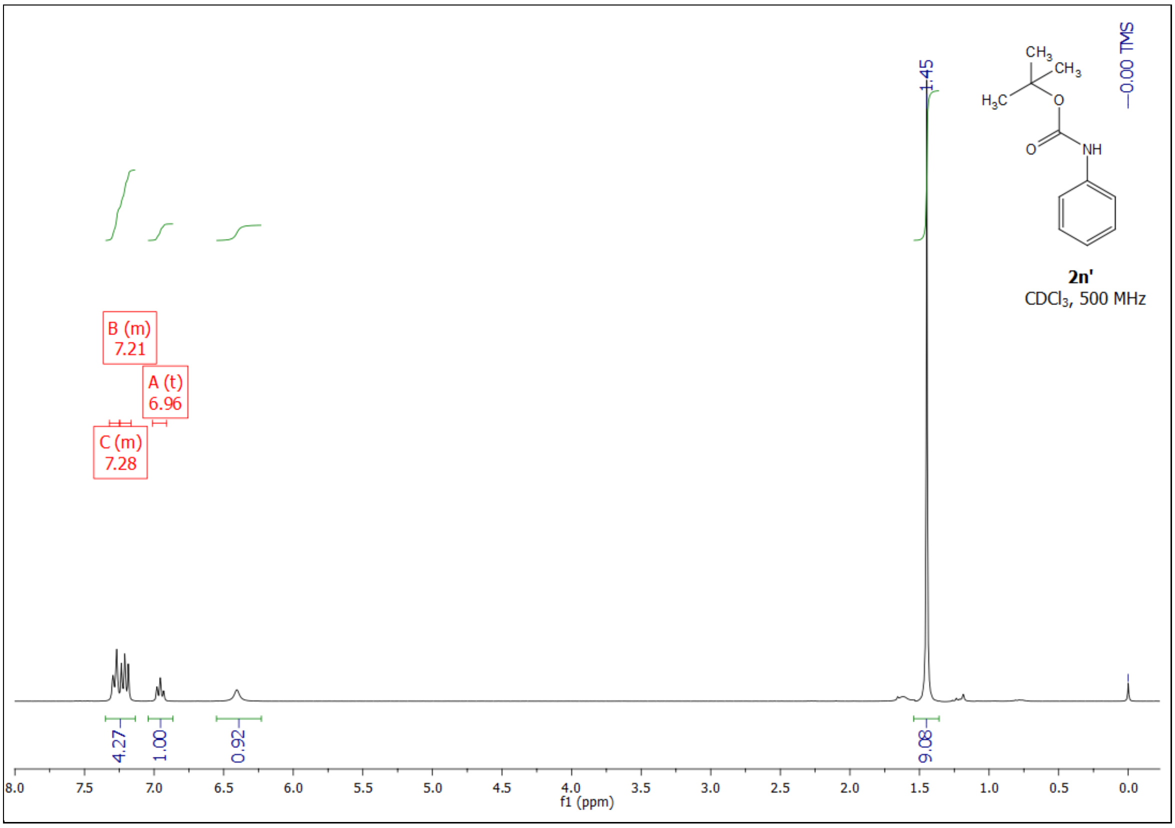

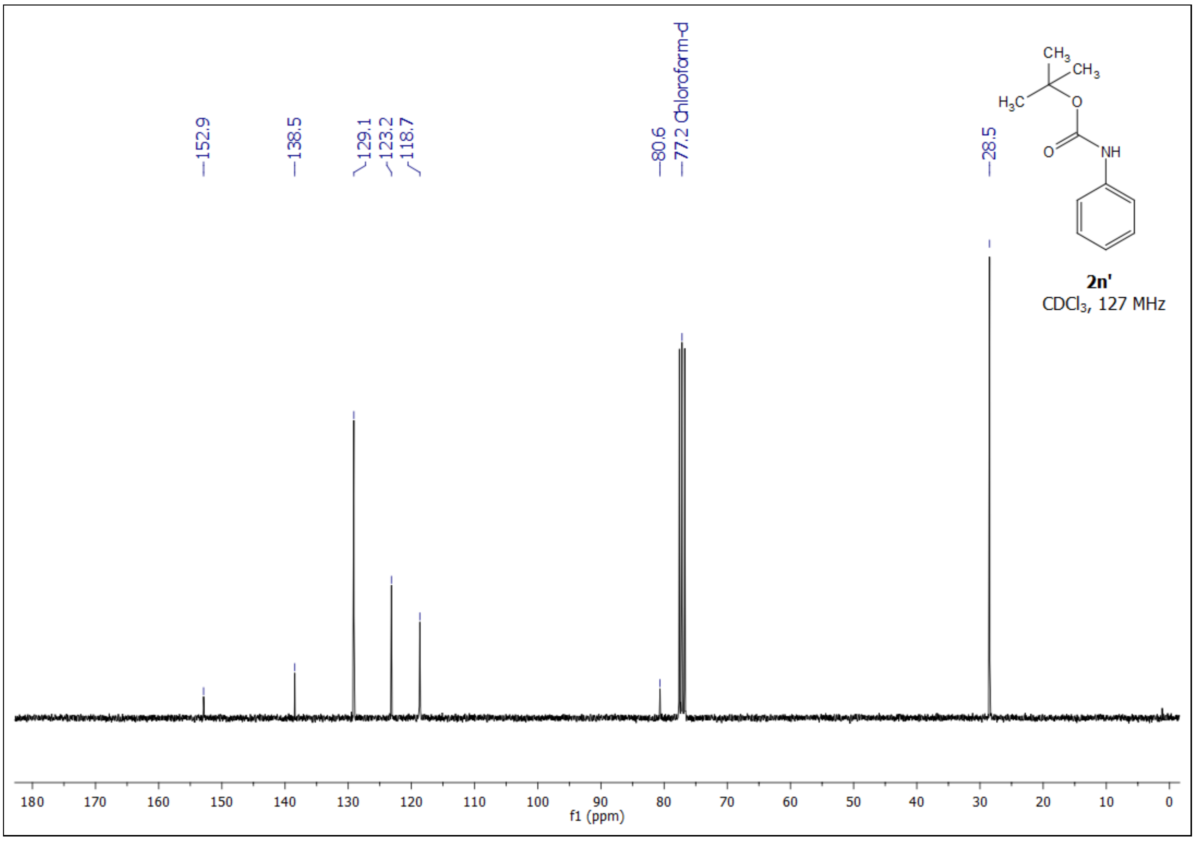

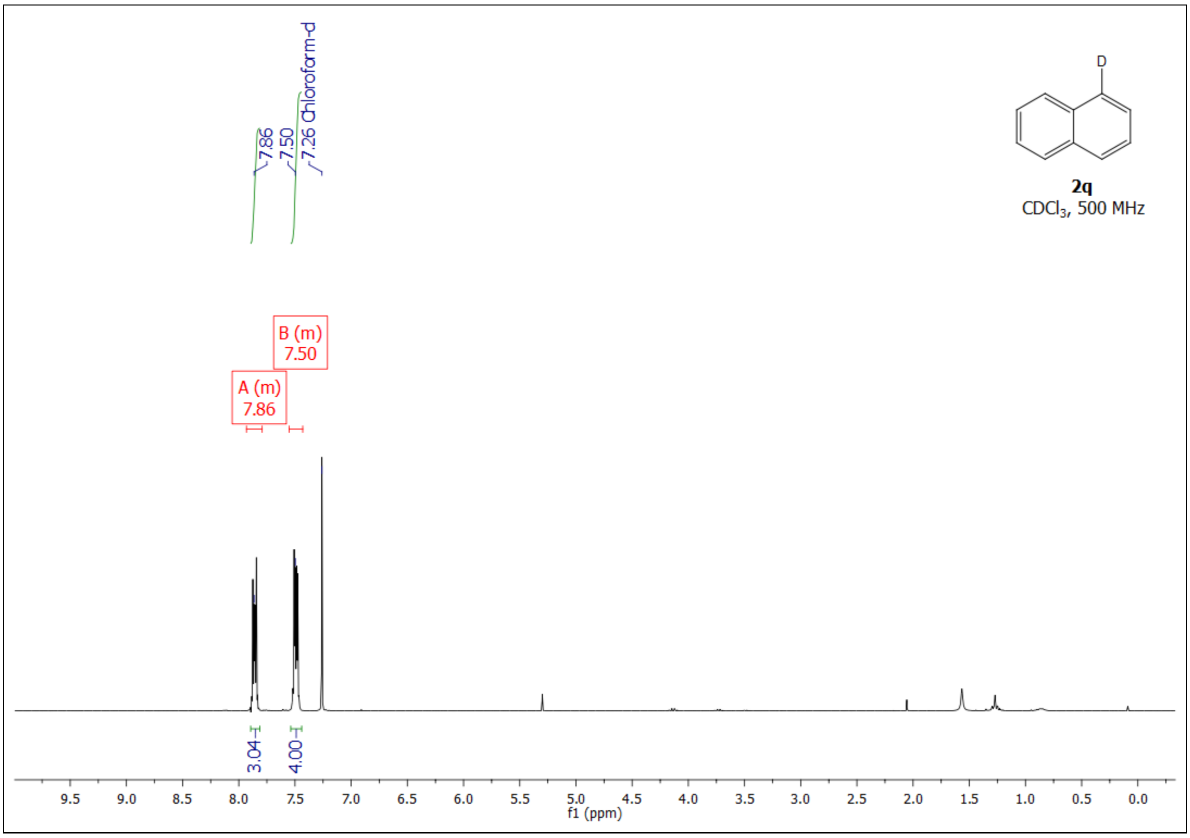

7.86
7.50
7.26 Chloroform-d
D
2q
CDCl3, 500 MHz
B (m)
7.50
A (m)
7.86
3.04
4.00
9.5
9.0
8.5
8.0
7.5
7.0
6.5
6.0
5.5
5.0
4.5
4.0
3.5
3.0
2.5
2.0
1.5
1.0
0.5
0.0
f1 (ppm)

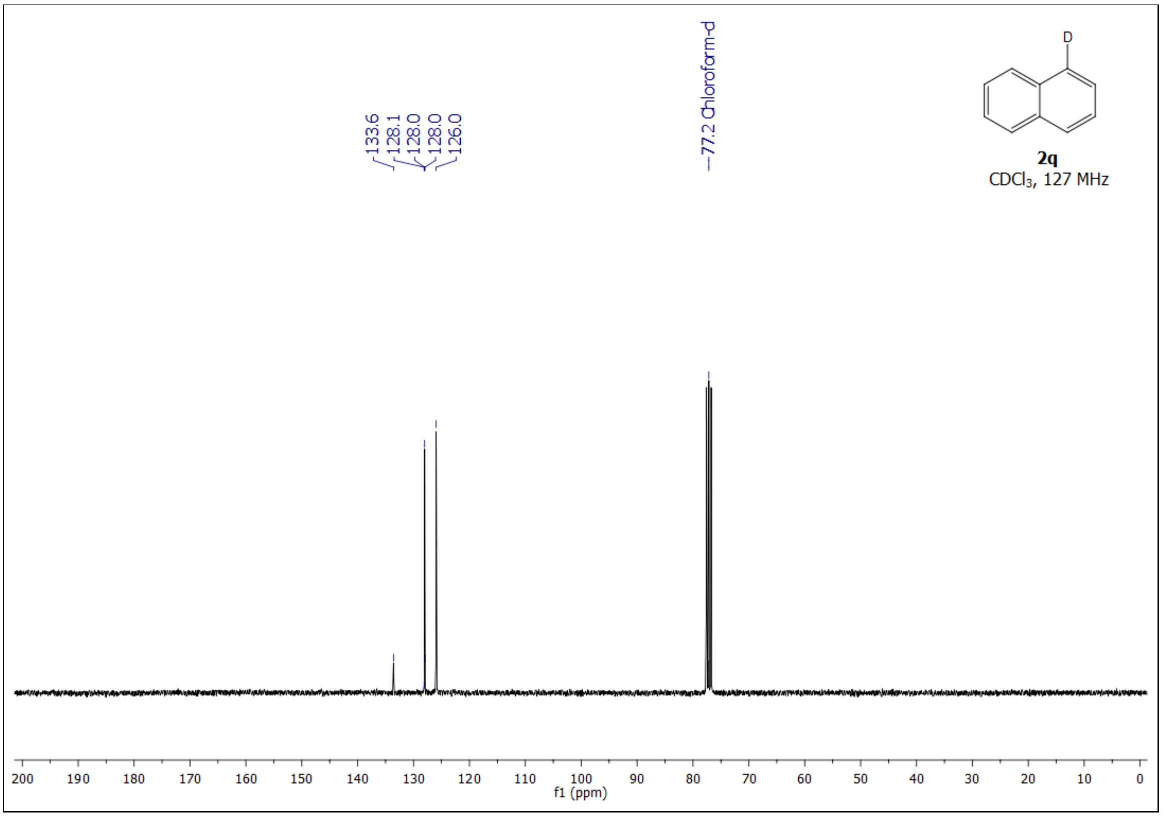

133.6
128.1
128.0
128.0
126.0
77.2 Chloroform-d
D
2q
CDCl3, 127 MHz
200
190
180
170
160
150
140
130
120
110
100
90
80
70
60
50
40
30
20
10
0
f1 (ppm)

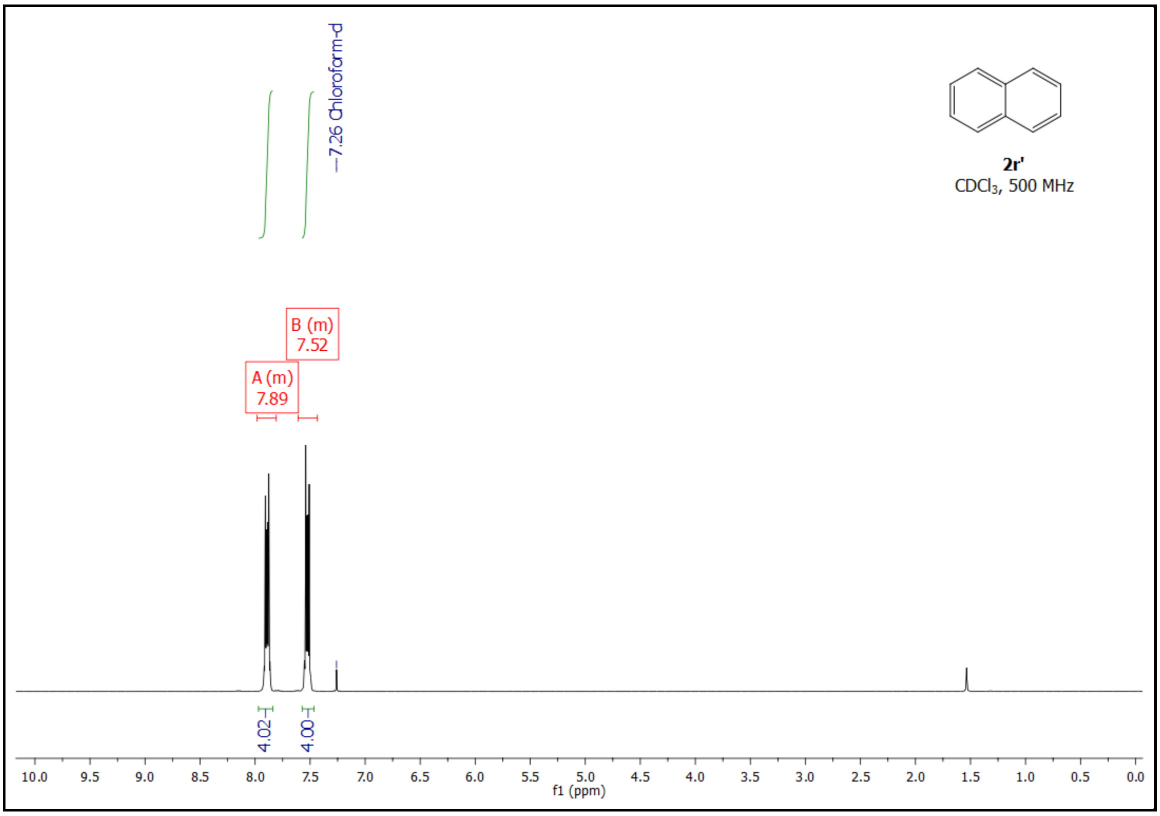
7.26 Chloroform-d
2r'
CDCl3, 500 MHz
B (m)
7.52
A (m)
7.89
4.02
4.00
10.0
9.5
9.0
8.5
8.0
7.5
7.0
6.5
6.0
5.5
5.0
4.5
4.0
3.5
3.0
2.5
2.0
1.5
1.0
0.5
0.0
f1 (ppm)

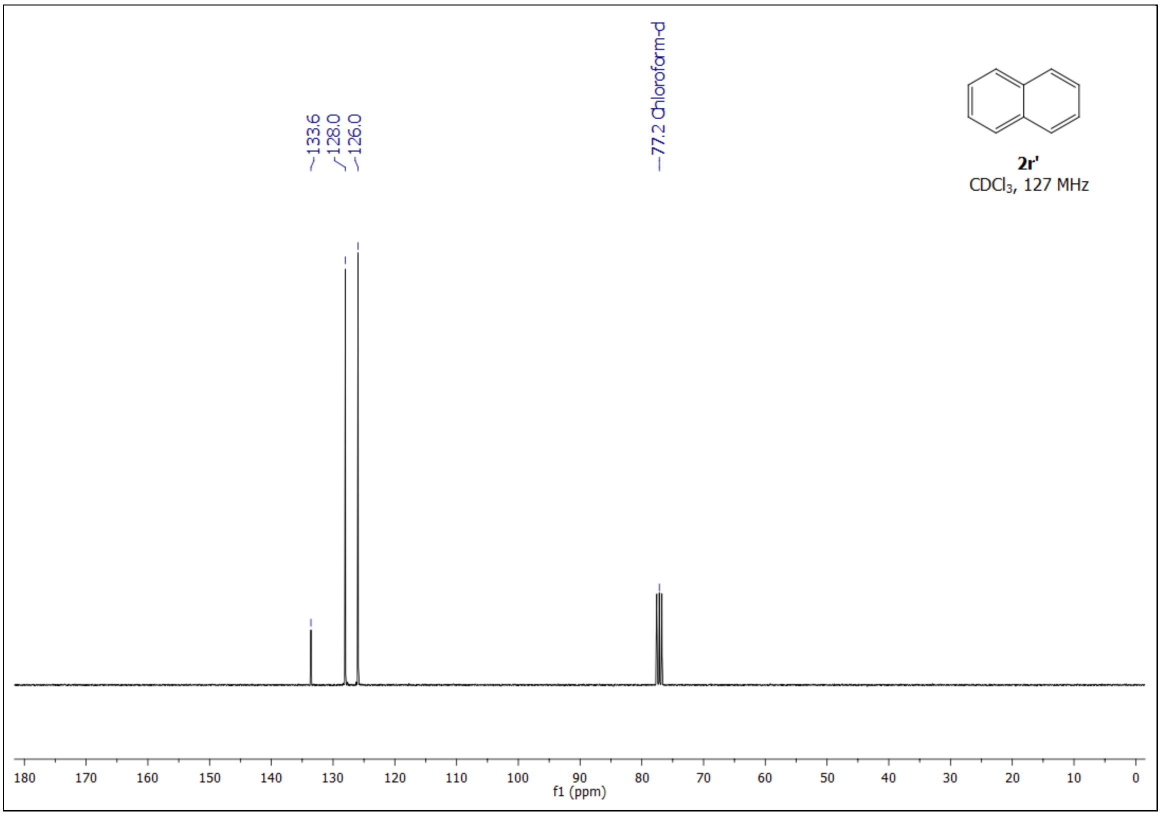
133.6
128.0
126.0
77.2 Chloroform-d
2r'
CDCl3, 127 MHz
180
170
160
150
140
130
120
110
100
90
80
70
60
50
40
30
20
10
0
f1 (ppm)

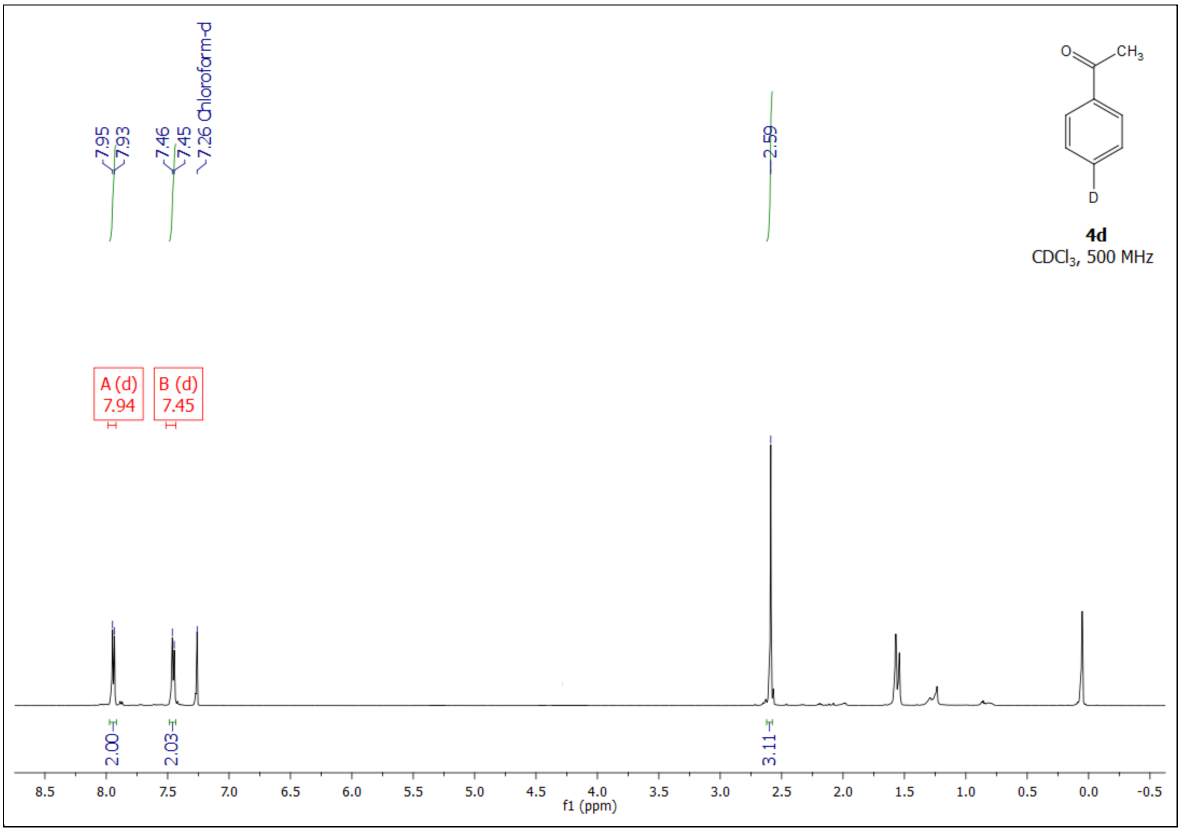

7.95
7.93
7.46
7.45
7.26 Chloroform-d
2.59
4d
CDCl3, 500 MHz
A (d) 7.94
B (d) 7.45
2.00
2.03
3.11
f1 (ppm)

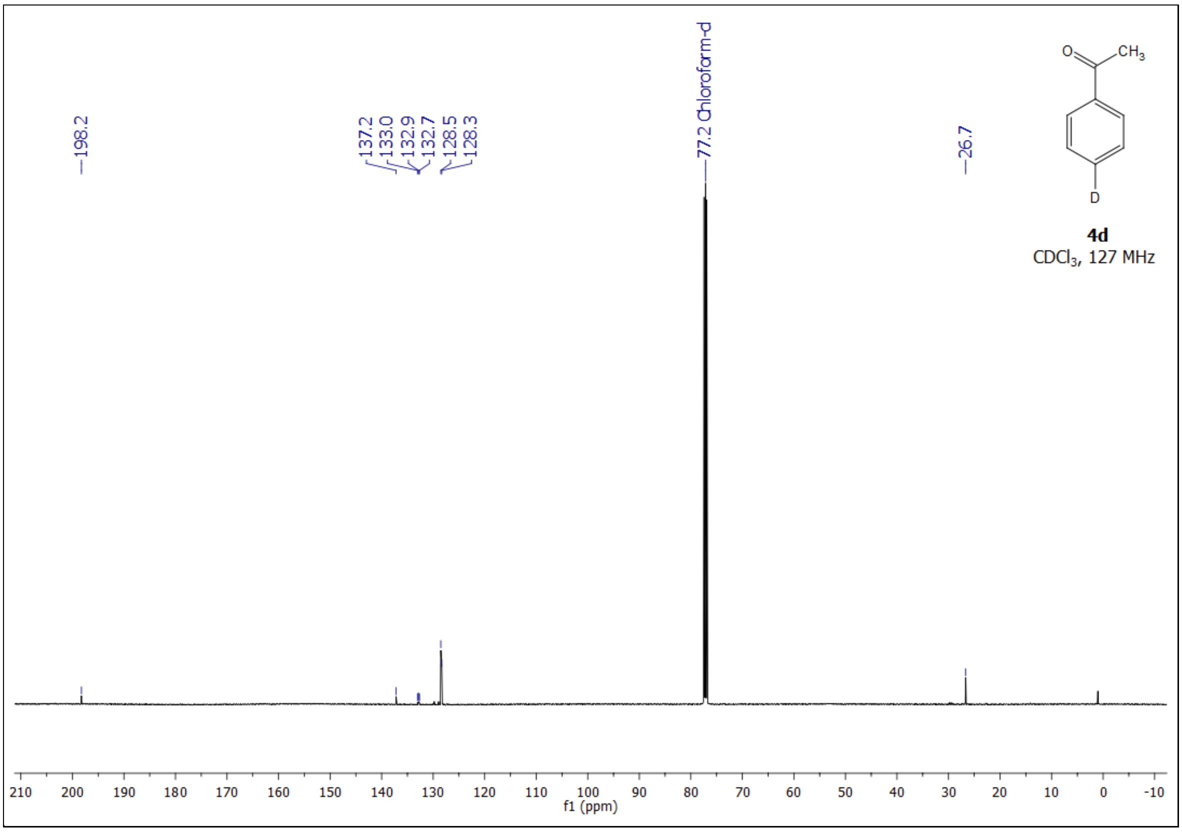

198.2
137.2
133.0
132.9
132.7
128.5
128.3
77.2 Chloroform-d
26.7
4d
CDCl3, 127 MHz
f1 (ppm)

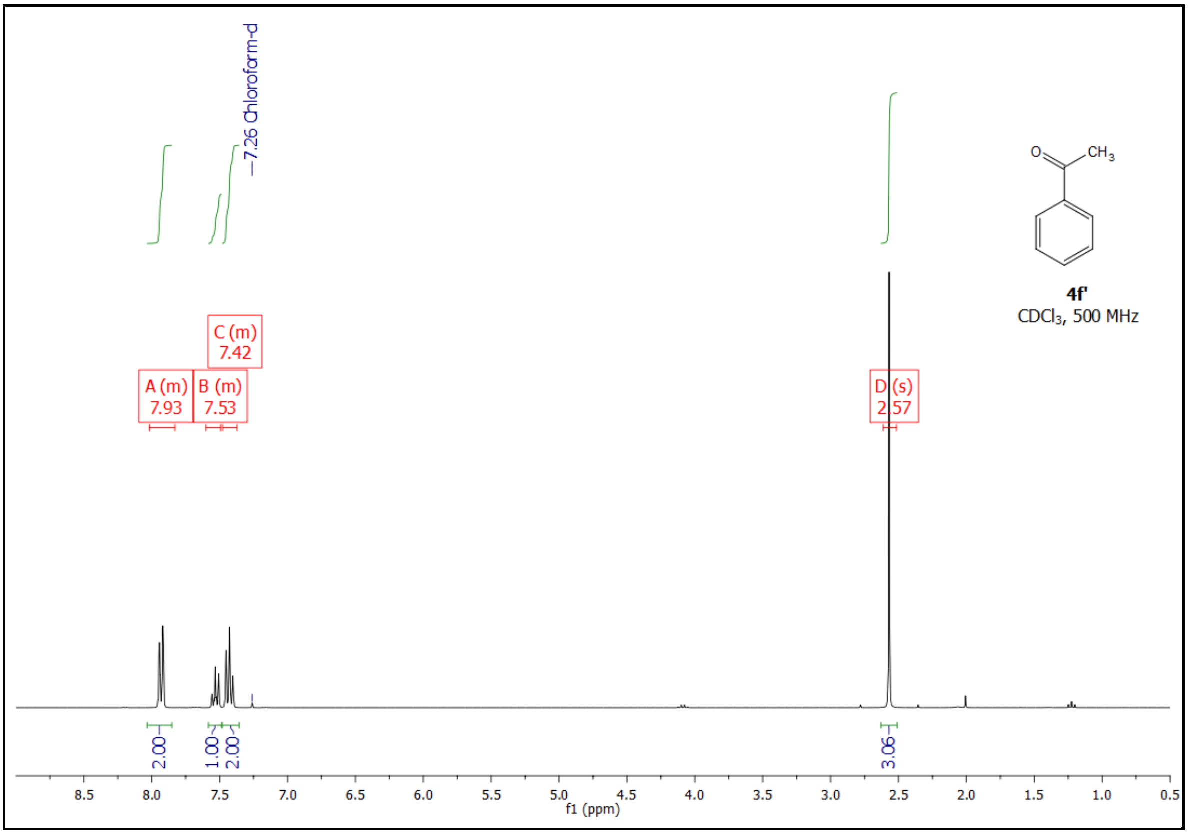
7.26 Chloroform-d
O
CH_3
4f'
$CDCl_3$, 500 MHz
C (m)
7.42
A (m)
7.93
B (m)
7.53
D (s)
2.57
2.00
1.00
2.00
3.06
8.5
8.0
7.5
7.0
6.5
6.0
5.5
5.0
4.5
4.0
3.5
3.0
2.5
2.0
1.5
1.0
0.5
f1 (ppm)

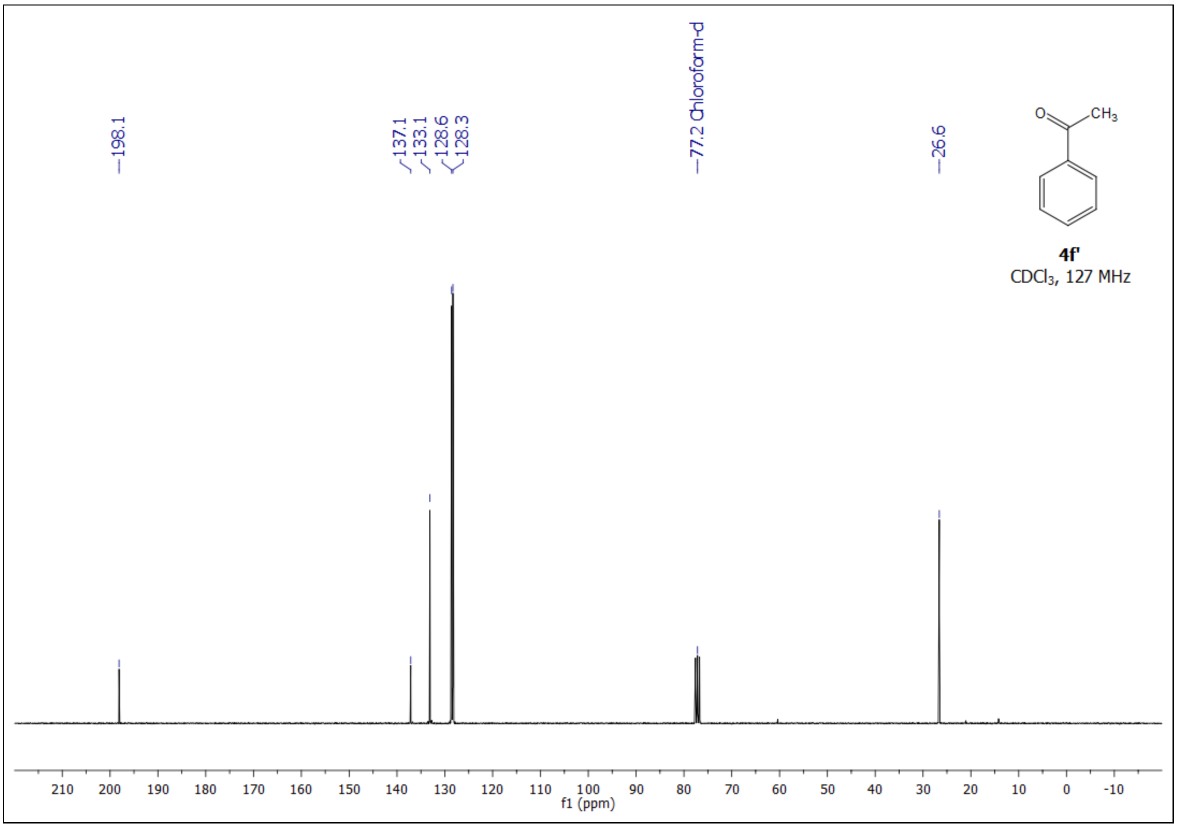
198.1
137.1
133.1
128.6
128.3
77.2 Chloroform-d
26.6
O
CH_3
4f'
$CDCl_3$, 127 MHz
210
200
190
180
170
160
150
140
130
120
110
100
90
80
70
60
50
40
30
20
10
0
-10
f1 (ppm)

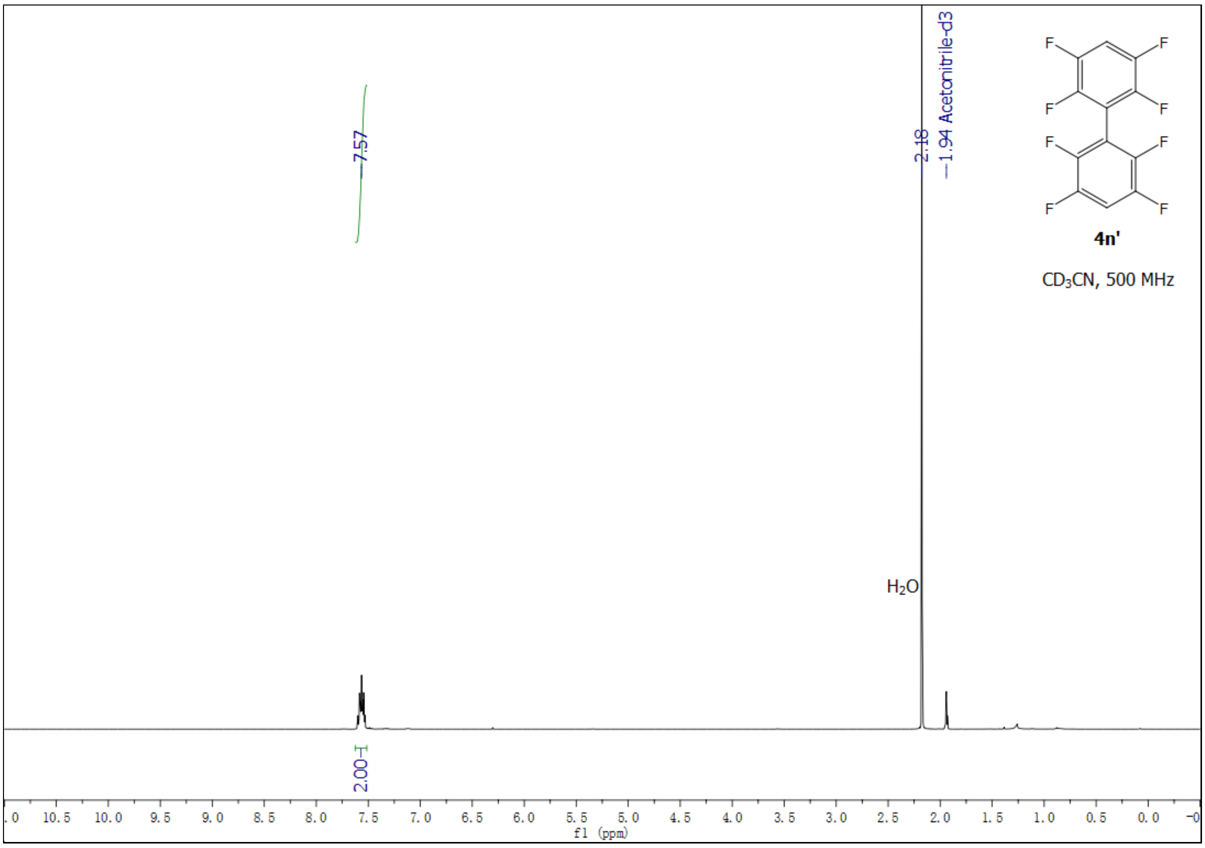
7.57
2.18
1.94 Acetonitrile-d3
H2O
2.00
4n'
CD3CN, 500 MHz
f1 (ppm)

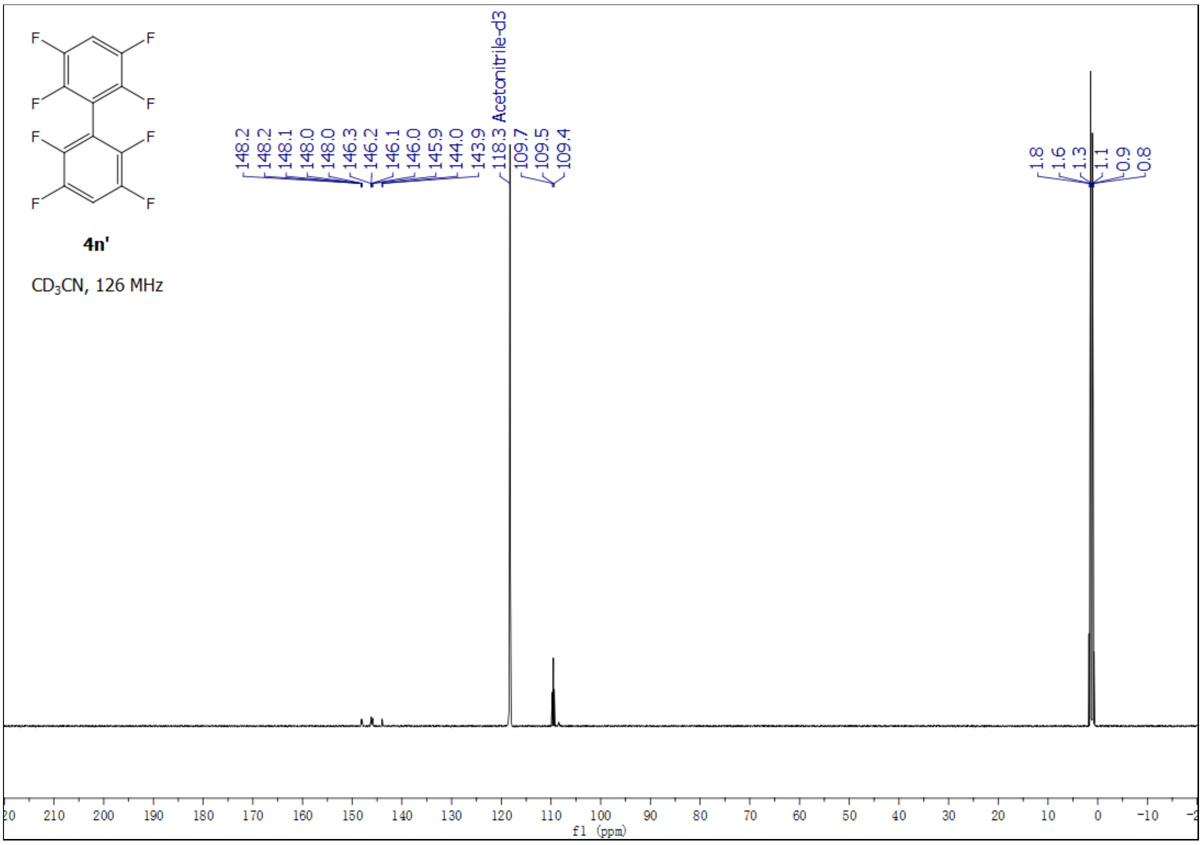
148.2
148.2
148.1
148.0
148.0
146.3
146.2
146.1
146.0
145.9
144.0
143.9
118.3 Acetonitrile-d3
109.7
109.5
109.4
1.8
1.6
1.3
1.1
0.9
0.8
4n'
CD3CN, 126 MHz
f1 (ppm)

References

[1] X. Lin, I. Telepeni, A. J. Blake, A. Dailly, C. M. Brown, J. M. Simmons, M. Zoppi, G. S. Walker, K. M. Thomas, T. J. Mays, P. Hubberstey, N. R. Champness, M. Schröder, *J. Am. Chem. Soc.* **2009**, *131*, 2159–2171.

[2] L. Briggs, R. Newby, X. Han, C. G. Morris, M. Savage, C. P. Krap, T. L. Easun, M. D. Frogley, G. Cinque, C. A. Murray, C. C. Tang, J. Sun, S. Yang, M. Schröder, *J. Mater. Chem. A* **2021**, *9*, 7190–7197.

[3] A. A. Coelho, *J. Appl. Crystallogr.* **2018**, *51*, 210–218.

[4] P. W. Stephens, *J. Appl. Crystallogr.* **1999**, *32*, 281–289.

[5] S. Stoll, A. Schweiger, *J. Magn. Reson.* **2006**, *178*, 42–55.

[6] B. C. Smith, *Spectroscopy* **2016**, *31*, 34–37.

[7] B. C. Smith, *Spectroscopy* **2017**, *32*, 28–34.

[8] B. C. Smith, *Spectroscopy* **2017**, *32*, 31–36.

[9] H. J. Li, R. Guillot, V. Gandon, *J. Org. Chem.* **2010**, *75*, 8435–8449.

[10] Y. Sawama, Y. Yabe, M. Shigetsura, T. Yamada, S. Nagata, Y. Fujiwara, T. Maegawa, Y. Monguchi, H. Sajiki, *Adv. Synth. Catal.* **2012**, *354*, 777–782.

[11] F. Otte, J. Kleinheider, W. Hiller, R. Wang, U. Englert, C. Strohmann, *J. Am. Chem. Soc.* **2021**, *143*, 4133–4137

[12] C. Liu, S. Han, M. Li, X. Chong, B. Zhang, *Angew. Chemie - Int. Ed.* **2020**, *59*, 18527–18531.

[13] A. V. Iosub, S. S. Stahl, *J. Am. Chem. Soc.* **2015**, *137*, 3454–3457.

[14] B. Yu, Y. Zhao, H. Zhang, J. Xu, L. Hao, X. Gao, Z. Liu, *Chem. Commun.* **2014**, *50*, 2330–2333.

[15] A. Wang, H. Jiang, *J. Org. Chem.* **2010**, *75*, 2321–2326.

[16] X. Jiang, J. Zhang, D. Zhao, Y. Li, *Chem. Commun.* **2019**, *55*, 2797–2800.

[17] A. Xia, X. Xie, X. Hu, W. Xu, Y. Liu, *J. Org. Chem.* **2019**, *84*, 13841–13857.

[18] S. Tang, M. Rauch, M. Montag, Y. Diskin-Posner, Y. Ben-David, D. Milstein, *J. Am. Chem. Soc.* **2020**, *142*, 20875–20882.

[19] G. Li, C. L. Ji, X. Hong, M. Szostak, *J. Am. Chem. Soc.* **2019**, *141*, 11161–11172.

[20] P. Boehm, S. Roediger, A. Bismuto, B. Morandi,. *Angew. Chem. Int. Ed.* **2020**, *59*, 17887-17896.

[21] G. Ramu, Y. Tangella, S. Ambala, B. Nagendra Babu, *J. Org. Chem.* **2020**, *85*, 5370–5378.

[22] S. Ghosh, A. Purkait, C. K. Jana, *Green Chem.* **2020**, *22*, 8721–8727.

[23] X. Xiong, Y. Y. Yeung, *Angew. Chemie - Int. Ed.* **2016**, *55*, 16101–16105.

[24] M. Janni, S. Peruncheralathan,. *Org. Biomol. Chem.* **2016**, *14*, 3091-3097.

[25] M. R. Adams, C. H. Tien, B. S. N. Huchenski, M. J. Ferguson, A. W. H. Speed, *Angew. Chemie - Int. Ed.* **2017**, *56*, 6268–6271.

[26] S. M. Senaweera, A. Singh, J. D. Weaver, *J. Am. Chem. Soc.* **2014**, *136*, 3002–3005.

[27] T. He, H. F. T. Klare, M.Oestreich, *J. Am. Chem. Soc.* **2022**, *144*, 4734-4738 (2022).

[28] C. Zarate, H. Yang, M. J. Bezdek, D. Hesk, P. J. Chirik, *J. Am. Chem. Soc.* **2019**, *141*, 5034–5044.

[29] A. A. Facundo, A. Arévalo, G. Fundora-Galano, M. Flores-Álamo, E. Orgaz, J. J. García, *New J. Chem.* **2019**, *43*, 6897–6908.

[30] A. C. Bissember, M. G. Banwell, *J. Org. Chem.* **2009**, *74*, 4893–4895.

[31] B. P. S. Chauhan, J. S. Rathore, T. Bandoo, *J. Am. Chem. Soc.* **2004**, *126*, 8493-8500.

[32] S. E. Baillie, T. D. Bluemke, W. Clegg, A. R. Kennedy, J. Klett, L. Russo, M. Tullio, E. Hevia, *Chem. Commun.* **2014**, *50*, 12859-12862.

[33] K. Chernichenko, M. Lindqvist, B. Kotai, M. Nieger, K. Sorochkina, I. Papai, T. Repo, *J. Am. Chem. Soc.* **2016**, *138*, 4860-4868.

[34] S. Shi, G. Meng, M. Szostak, *Angew. Chem. Int. Ed.* **2006**, *55*, 6959-6963.

[35] S. D. Schimler, M. A. Cismesia, P. S. Hanley, R. D. J. Froese, M. J. Jansma, D. C. Bland, M. S. Sanford, *J. Am. Chem. Soc.* **2017**, *139*, 1452–1455.

[36] C. A. Malapit, I. K. Luvaga, J. T. Reeves, I. Volchkov, C. A. Busacca, A. R. Howell, C. H. Senanayake, *J. Org. Chem.* **2017**, *82*, 4993–4997.

[37] Y. Pang, M. Leutzsch, N. Nöthling, F. Katzenburg, C. Josep, *J. Am. Chem. Soc.* **2021**, *143*,

12487-12493.

[38] Y. Pang, M. Leutzsch, N. Nöthling, F. Katzenburg, J. Cornella, *J. Am. Chem. Soc.* **2021**, *143*, 12487–12493

[39] Y. Liu, S. Liu, D. Li, N. Zhang, L. Peng, J. Ao, C. E. Song, Y. Lan, H. Yan, *J. Am. Chem. Soc.* **2019**, *141*, 1150–1159.

[40] P. Mizar, T. Wirth, *Angew. Chem. Int. Ed.* **2014**, *53*, 5993-5997.
